# Supplementary figures and images for: Dynamic regulation of mRNA acetylation at synapses by spatial memory in mouse hippocampus
Source: eLife. 2026 Mar 23;14:RP108995. doi: 10.7554/eLife.108995 (PMC13008358; doi:10.7554/eLife.108995)

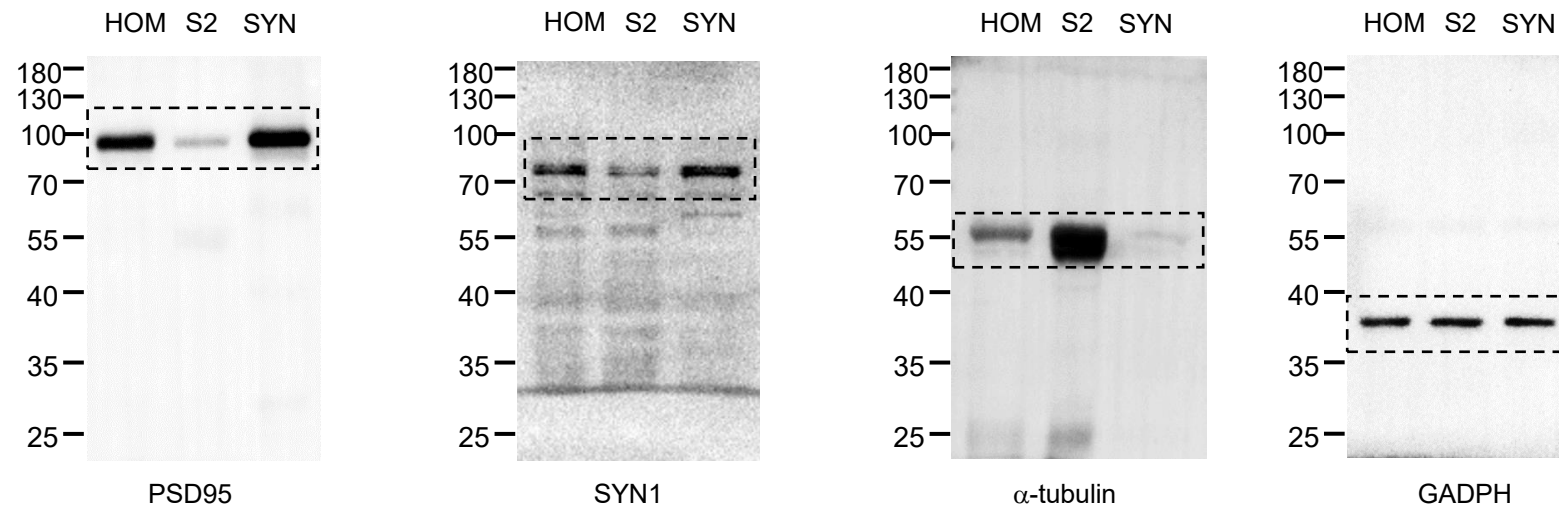

**Figure 2, Source Data 1.** Original membranes corresponding to Figure 2, panel A.

Supplement: Figure 2—source data 1. [file elife-108995-fig2-data1.zip › Figure 2, Source Data 1.pdf]

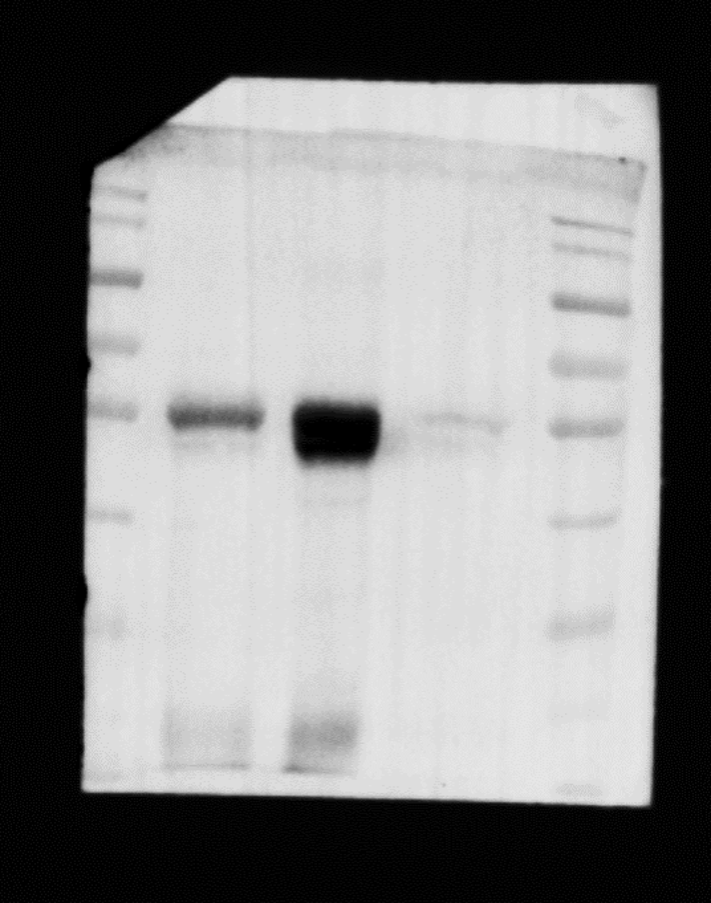

Supplement: Figure 2—source data 2. [file elife-108995-fig2-data2.zip › Figure 2, Source Data 2/a-tubulin.tif]

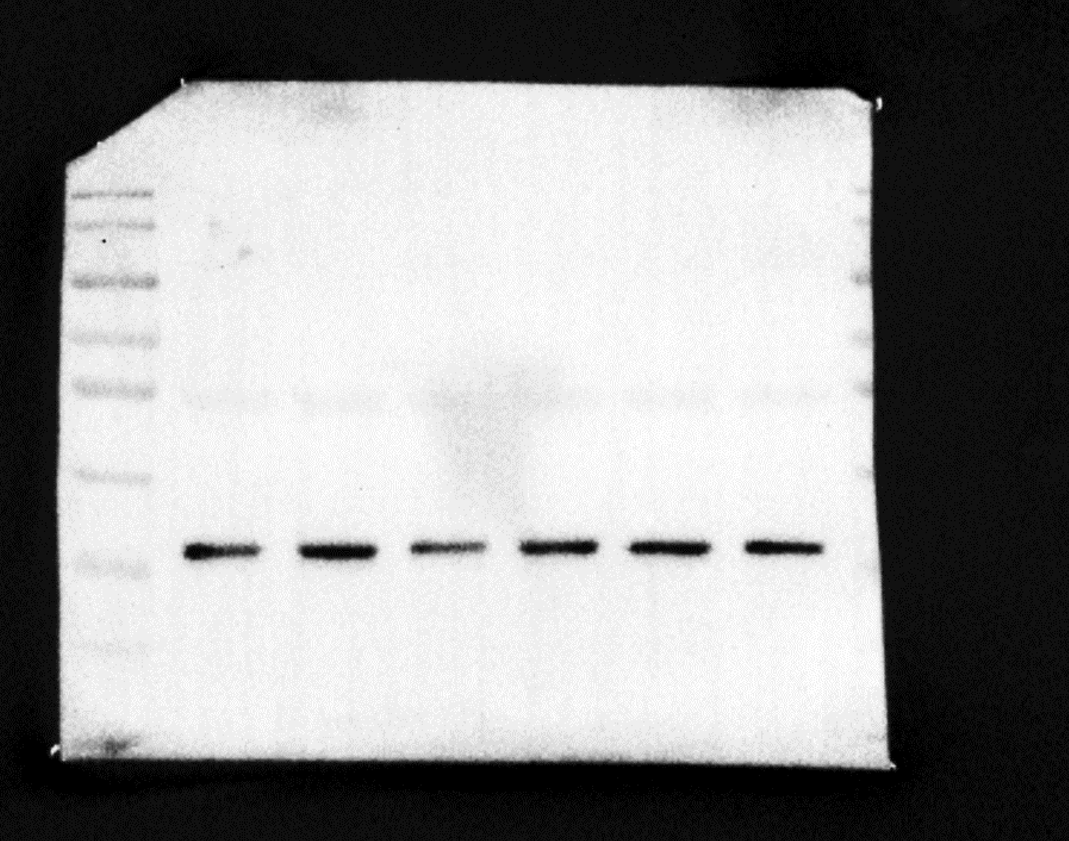

Supplement: Figure 2—source data 2. [file elife-108995-fig2-data2.zip › Figure 2, Source Data 2/GADPH.tif]

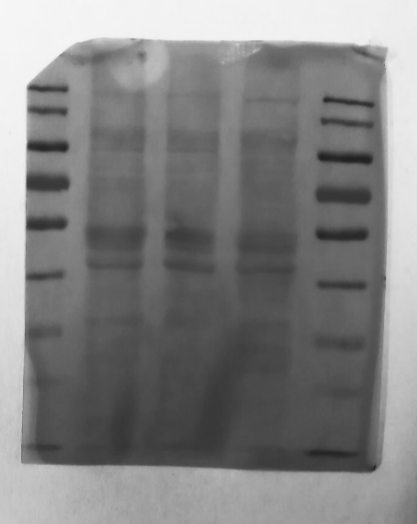

Supplement: Figure 2—source data 2. [file elife-108995-fig2-data2.zip › Figure 2, Source Data 2/Ponceau staining.tif]

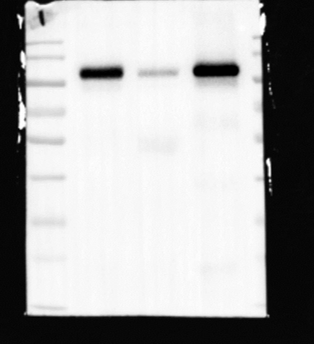

Supplement: Figure 2—source data 2. [file elife-108995-fig2-data2.zip › Figure 2, Source Data 2/PSD95.tif]

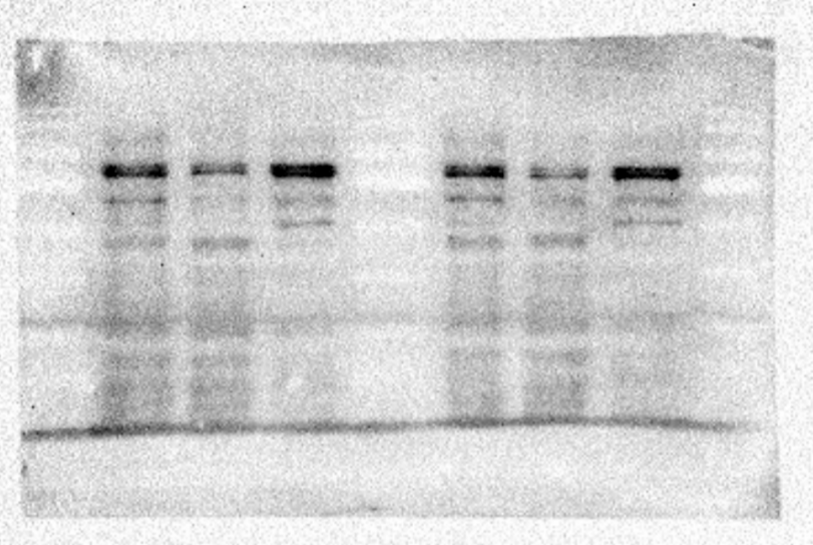

Supplement: Figure 2—source data 2. [file elife-108995-fig2-data2.zip › Figure 2, Source Data 2/SYN1.tif]

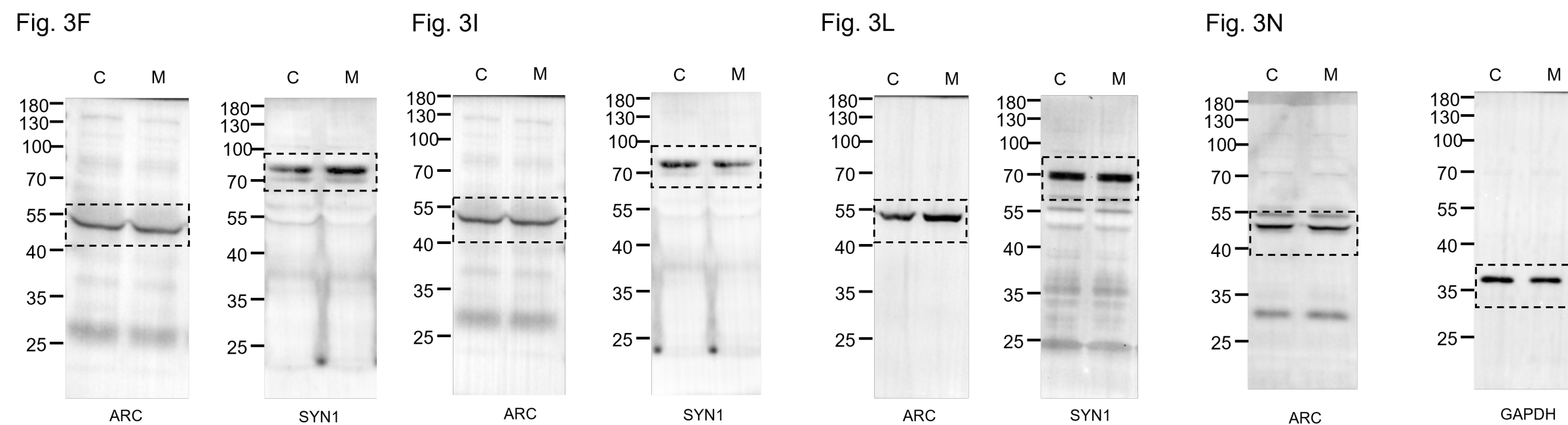

**Figure 3, Source Data 1.** Original membranes corresponding to Figure 3, panel F, I, L, N.

Supplement: Figure 3—source data 1. [file elife-108995-fig3-data1.zip › Figure 3, Source Data 1.pdf]

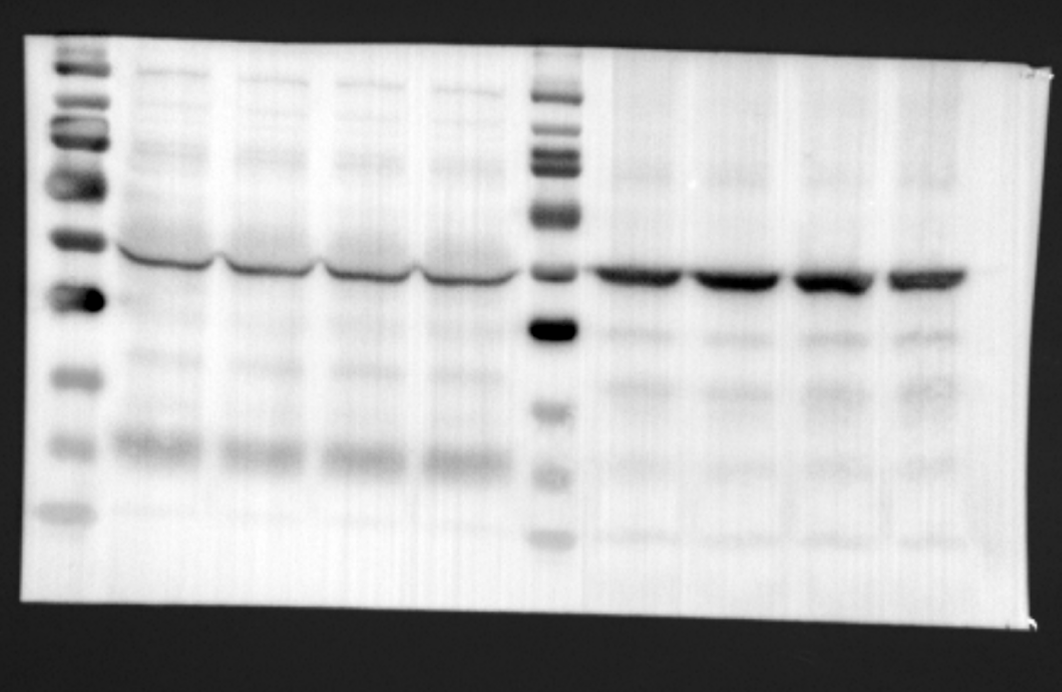

Supplement: Figure 3—source data 2. [file elife-108995-fig3-data2.zip › Figure 3, Source Data 2/ARC (F and I).tif]

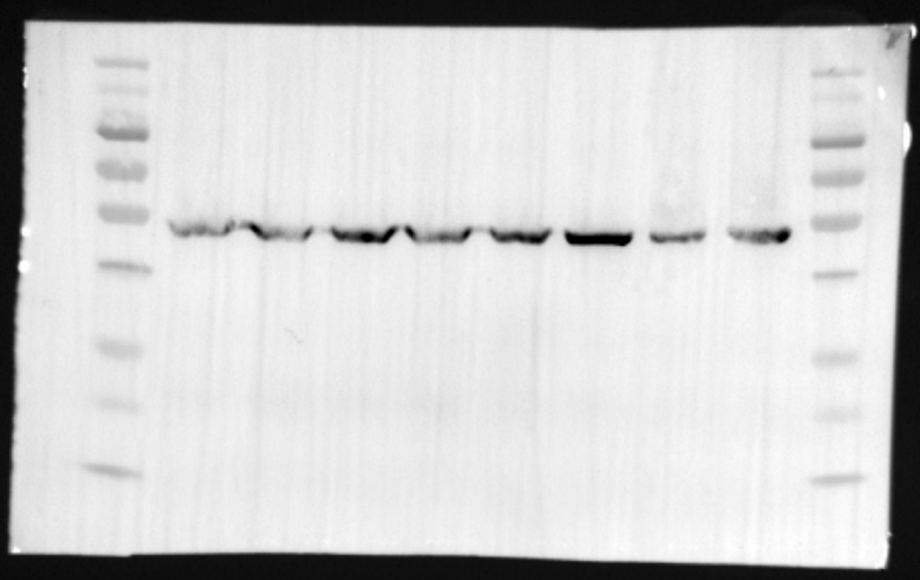

Supplement: Figure 3—source data 2. [file elife-108995-fig3-data2.zip › Figure 3, Source Data 2/ARC (L).tif]

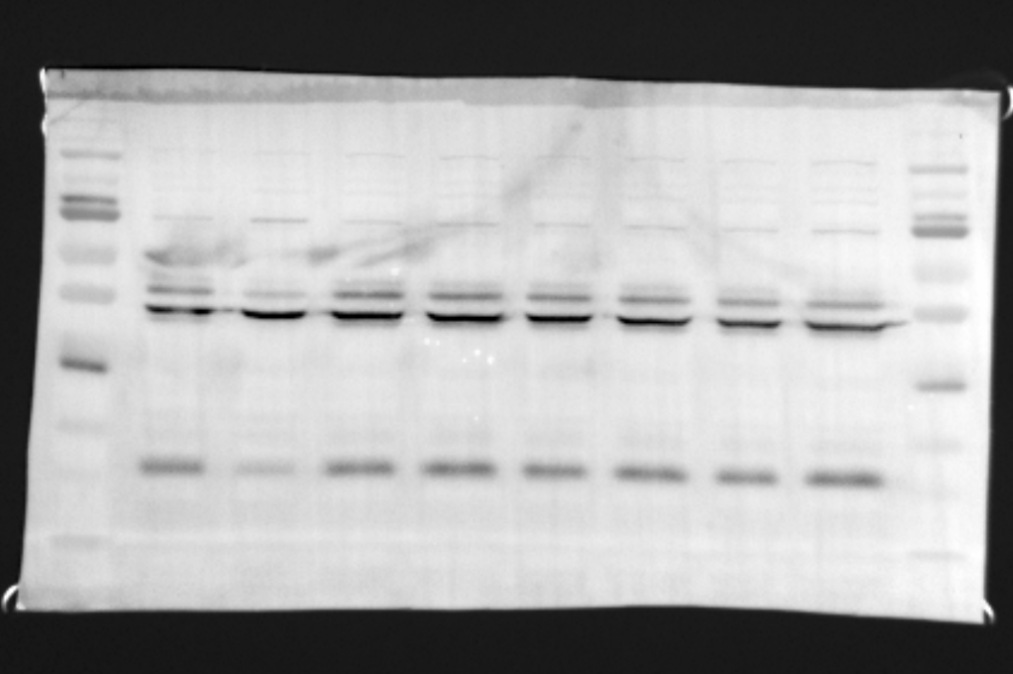

Supplement: Figure 3—source data 2. [file elife-108995-fig3-data2.zip › Figure 3, Source Data 2/ARC (N).tif]

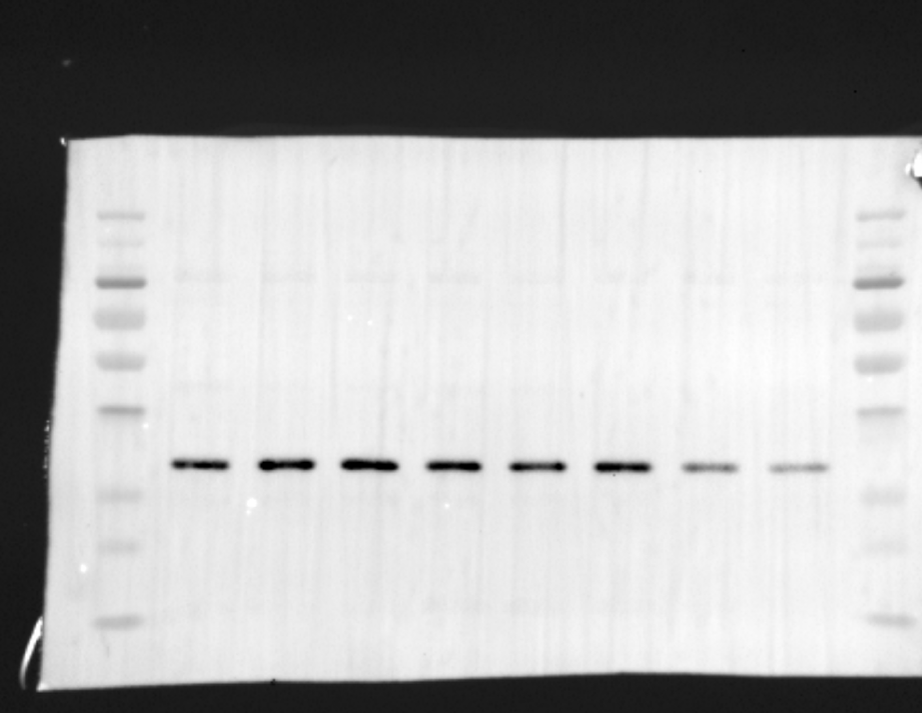

Supplement: Figure 3—source data 2. [file elife-108995-fig3-data2.zip › Figure 3, Source Data 2/GAPDH (N).tif]

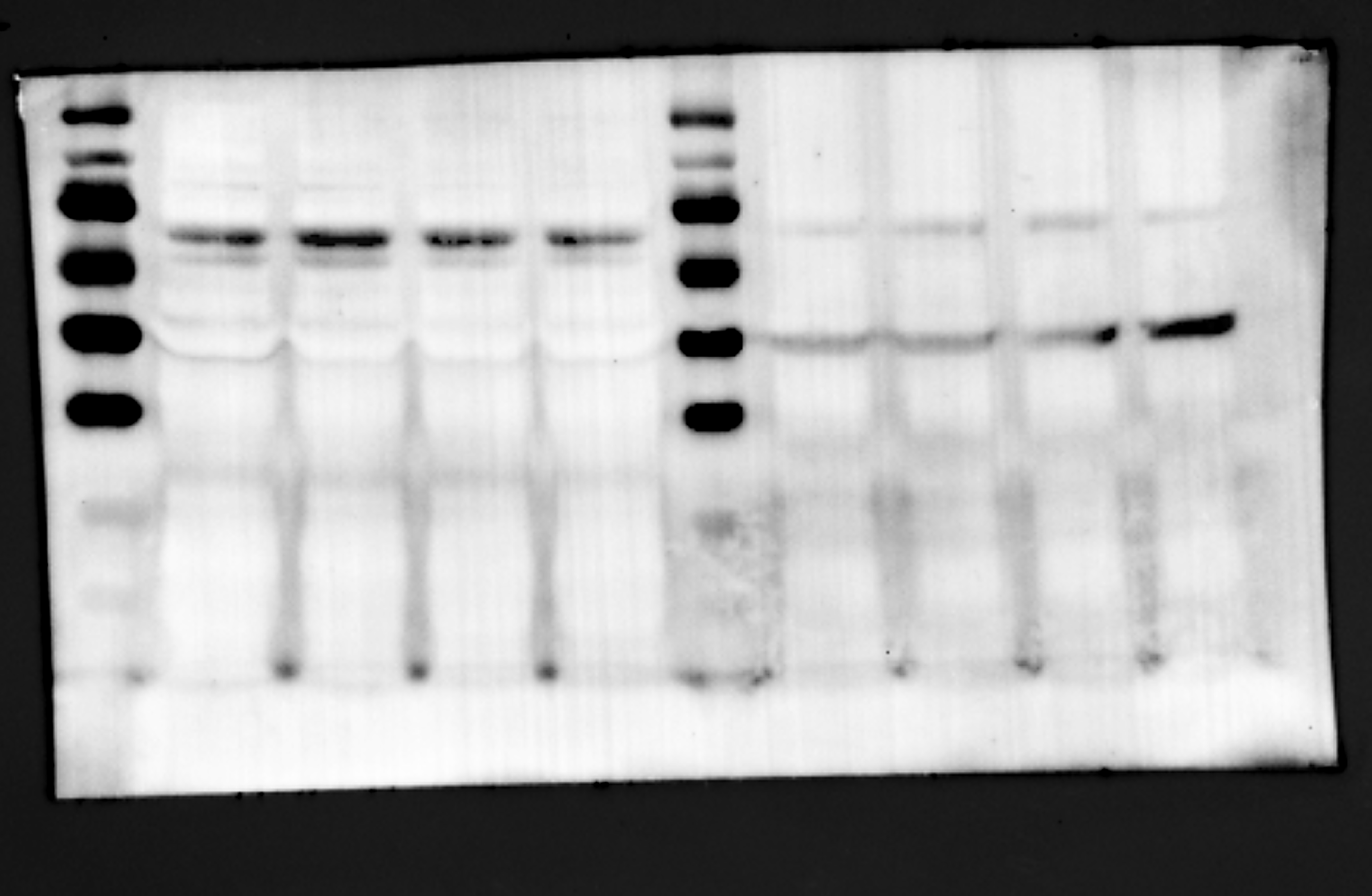

Supplement: Figure 3—source data 2. [file elife-108995-fig3-data2.zip › Figure 3, Source Data 2/SYN1 (F and I).tif]

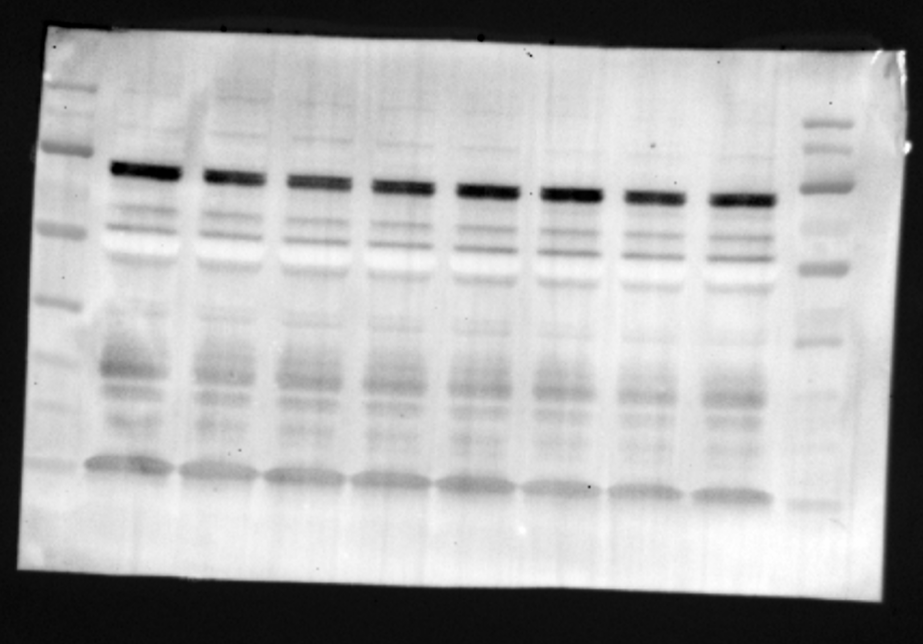

Supplement: Figure 3—source data 2. [file elife-108995-fig3-data2.zip › Figure 3, Source Data 2/SYN1 (L).tif]

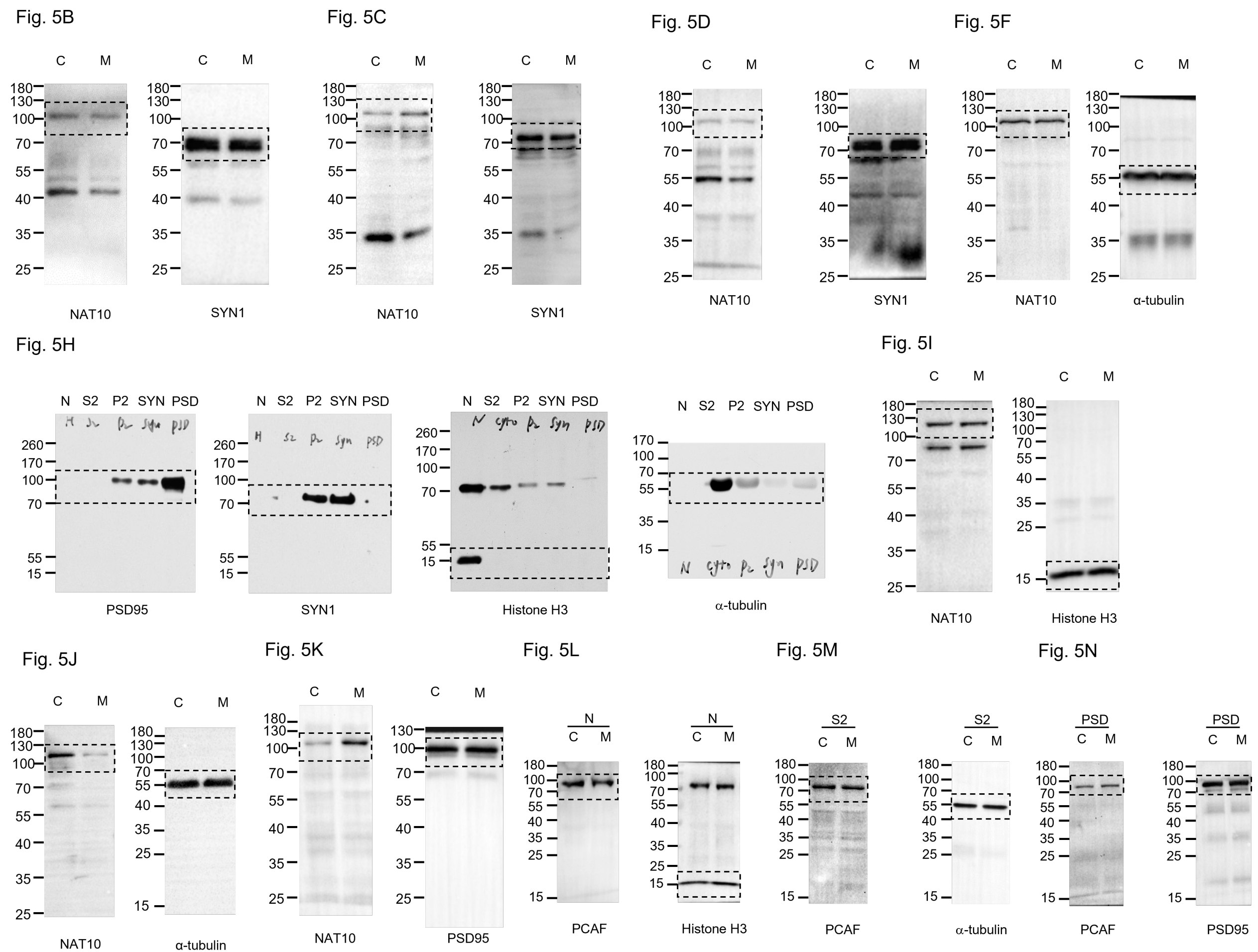

**Figure 5, Source Data 1.** Original membranes corresponding to Figure 5.

Supplement: Figure 5—source data 1. [file elife-108995-fig5-data1.zip › Figure 5, Source Data 1.pdf]

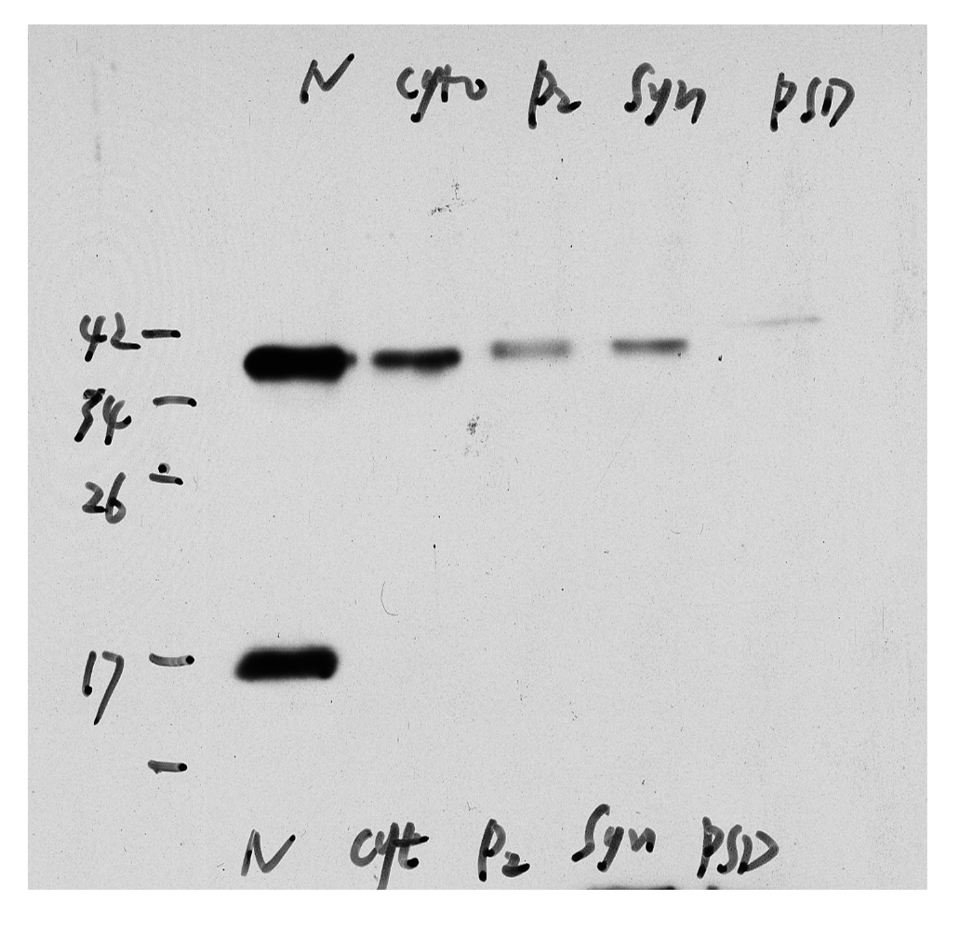

Supplement: Figure 5—source data 2. [file elife-108995-fig5-data2.zip › Figure 5, Source Data 2/Histone H3 (H).tif]

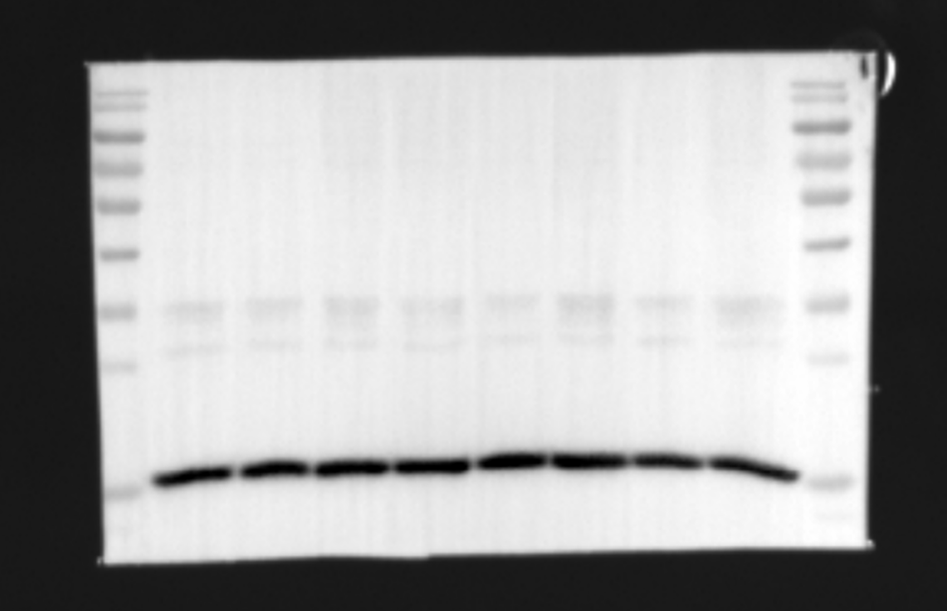

Supplement: Figure 5—source data 2. [file elife-108995-fig5-data2.zip › Figure 5, Source Data 2/Histone H3 (I).tif]

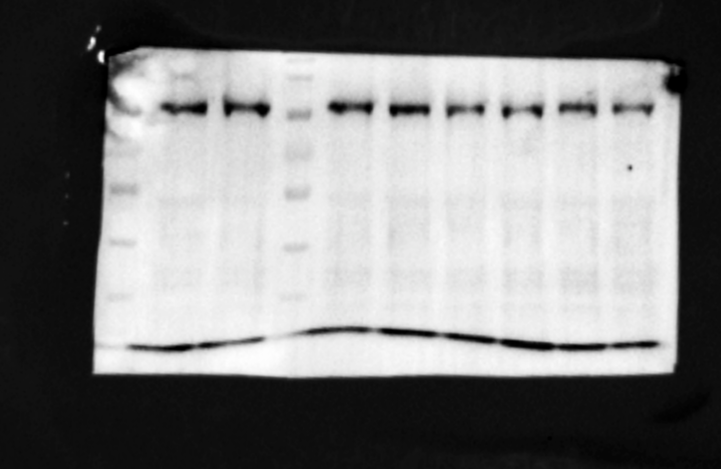

Supplement: Figure 5—source data 2. [file elife-108995-fig5-data2.zip › Figure 5, Source Data 2/Histone H3 (L).tif]

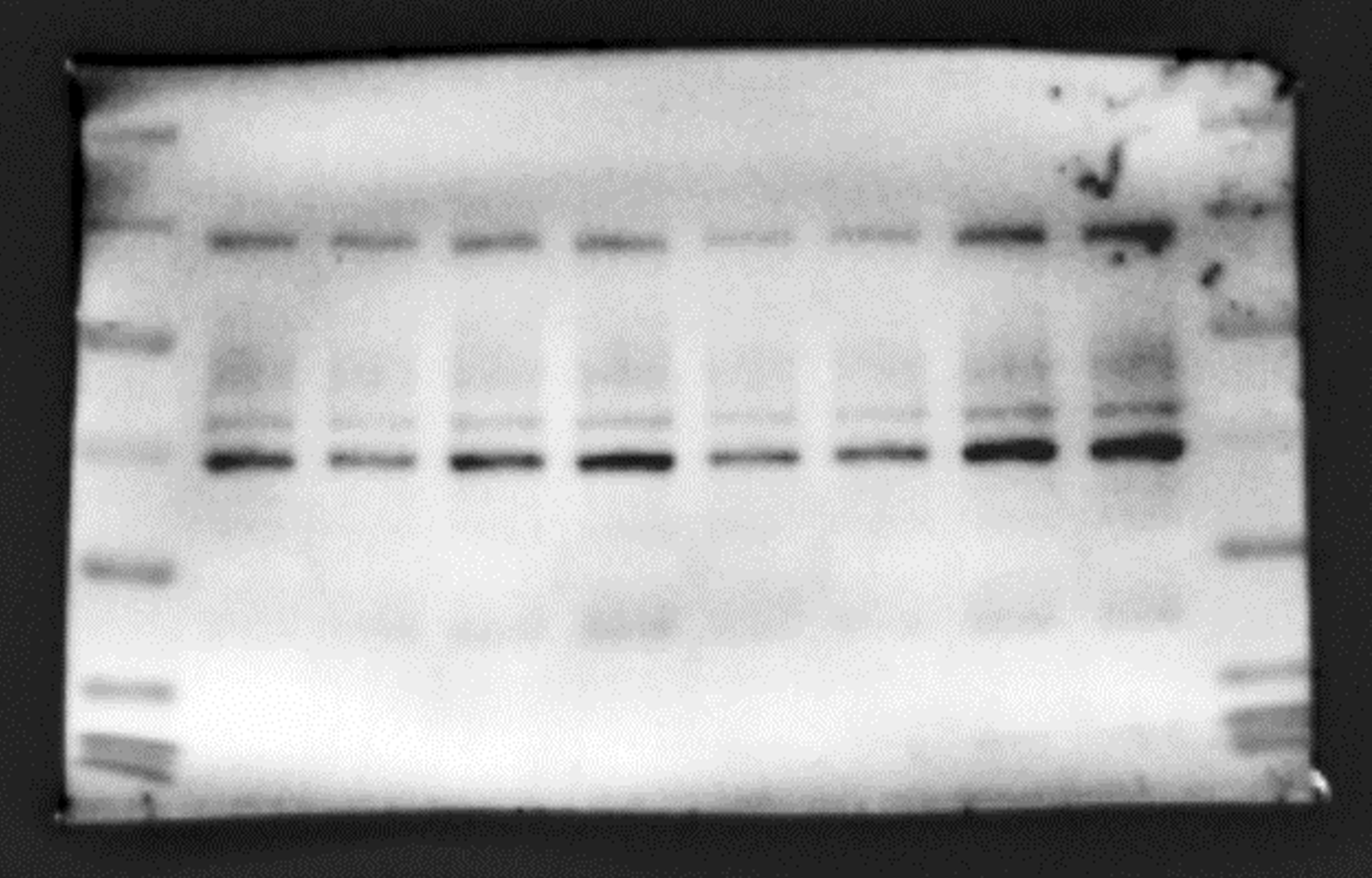

Supplement: Figure 5—source data 2. [file elife-108995-fig5-data2.zip › Figure 5, Source Data 2/NAT10 (B).tif]

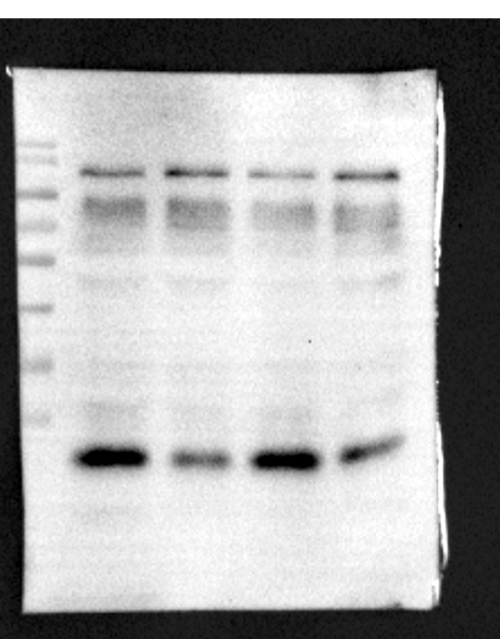

Supplement: Figure 5—source data 2. [file elife-108995-fig5-data2.zip › Figure 5, Source Data 2/NAT10 (C).tif]

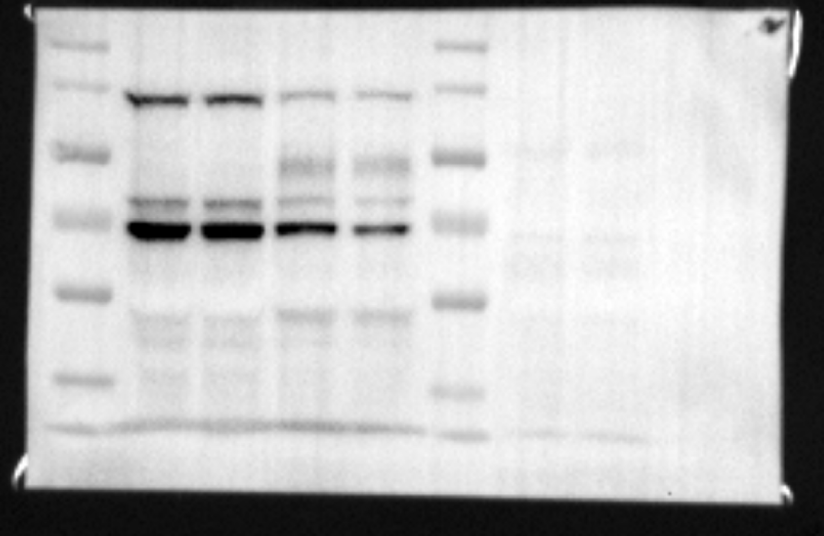

Supplement: Figure 5—source data 2. [file elife-108995-fig5-data2.zip › Figure 5, Source Data 2/NAT10 (D).tif]

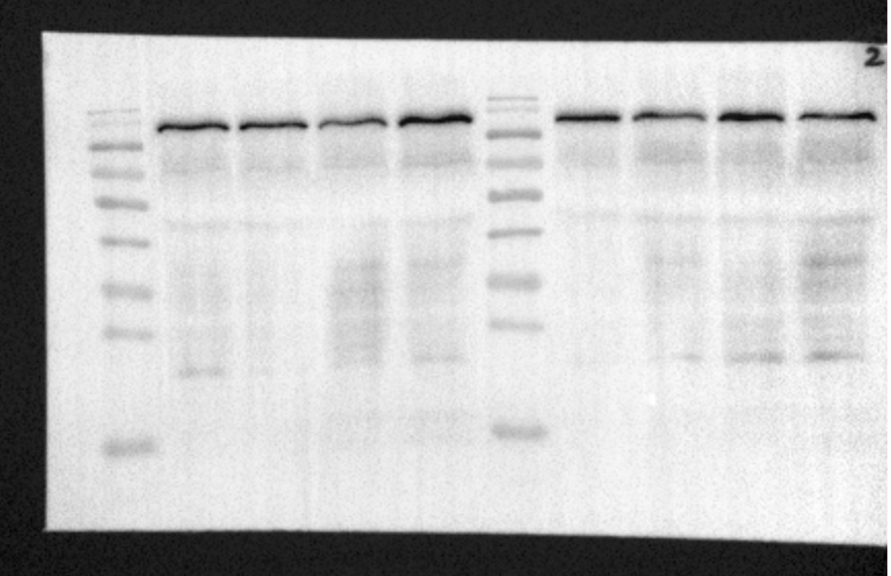

Supplement: Figure 5—source data 2. [file elife-108995-fig5-data2.zip › Figure 5, Source Data 2/NAT10 (F).tif]

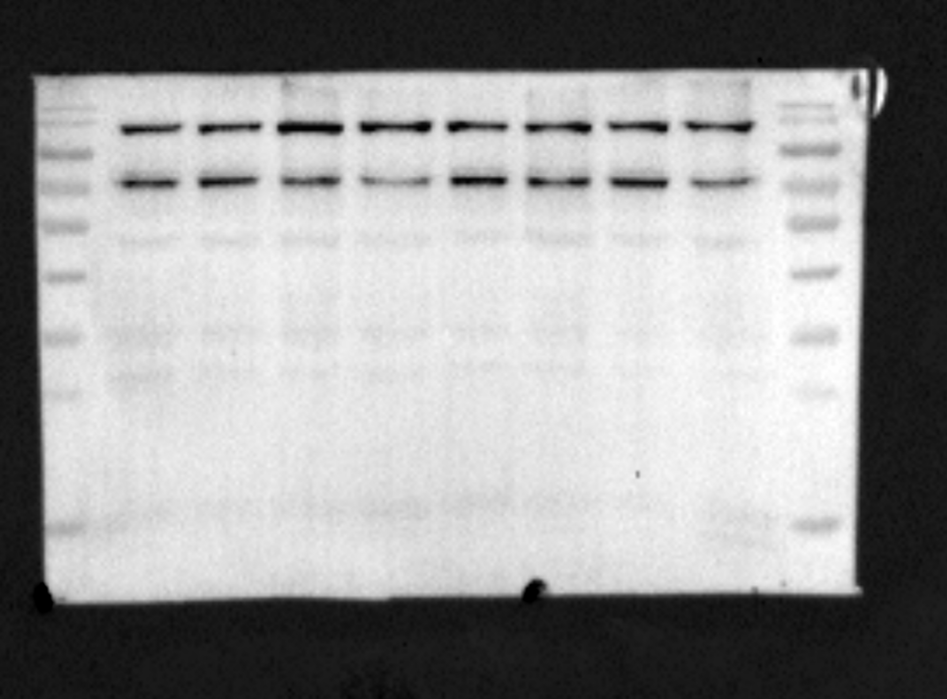

Supplement: Figure 5—source data 2. [file elife-108995-fig5-data2.zip › Figure 5, Source Data 2/NAT10 (I).tif]

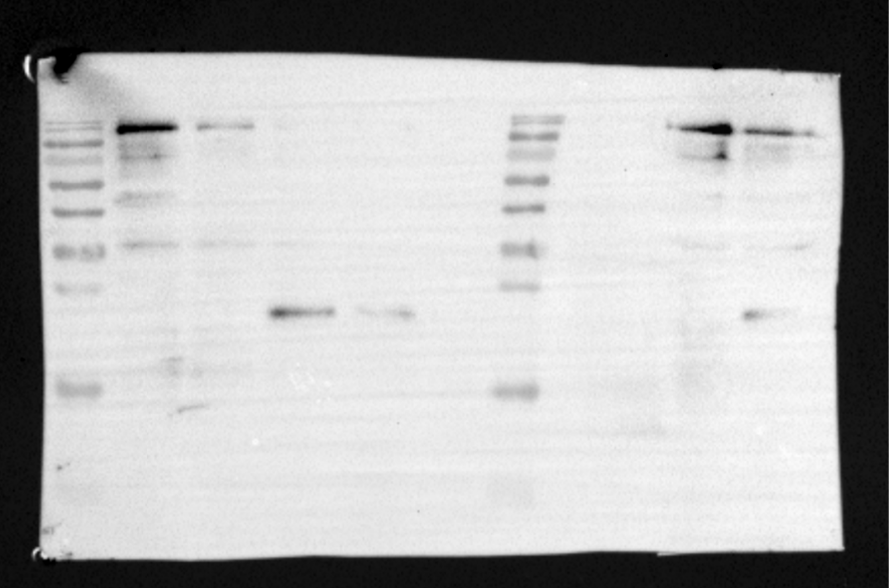

Supplement: Figure 5—source data 2. [file elife-108995-fig5-data2.zip › Figure 5, Source Data 2/NAT10 (J).tif]

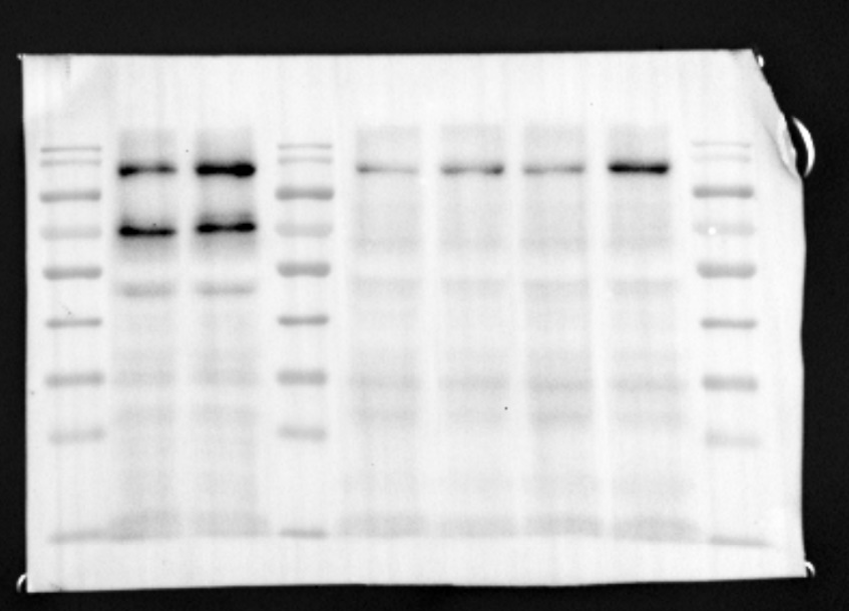

Supplement: Figure 5—source data 2. [file elife-108995-fig5-data2.zip › Figure 5, Source Data 2/NAT10 (K).tif]

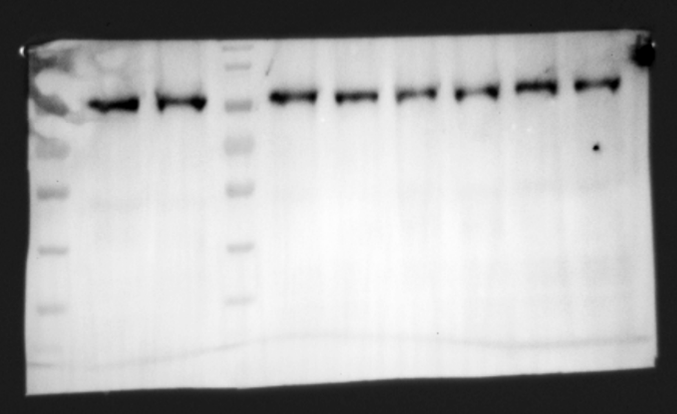

Supplement: Figure 5—source data 2. [file elife-108995-fig5-data2.zip › Figure 5, Source Data 2/PCAF (L).tif]

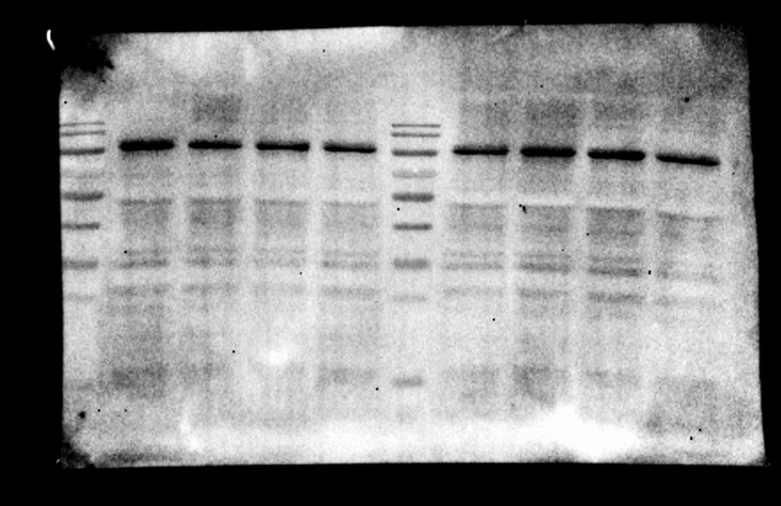

Supplement: Figure 5—source data 2. [file elife-108995-fig5-data2.zip › Figure 5, Source Data 2/PCAF (M).tif]

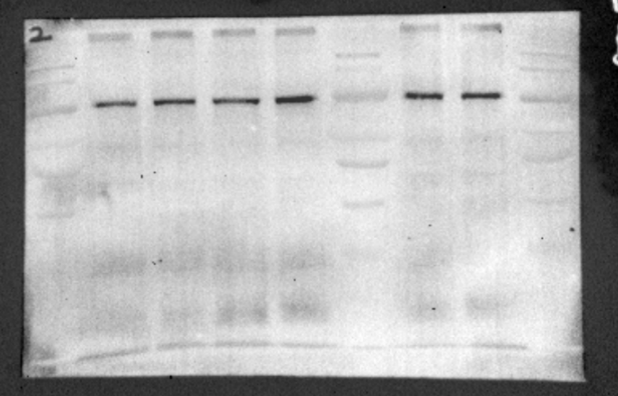

Supplement: Figure 5—source data 2. [file elife-108995-fig5-data2.zip › Figure 5, Source Data 2/PCAF (N).tif]

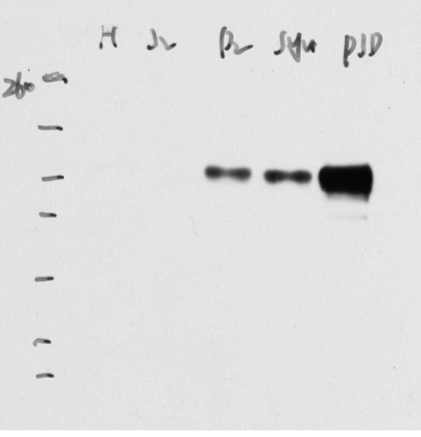

Supplement: Figure 5—source data 2. [file elife-108995-fig5-data2.zip › Figure 5, Source Data 2/PSD95 (H).tif]

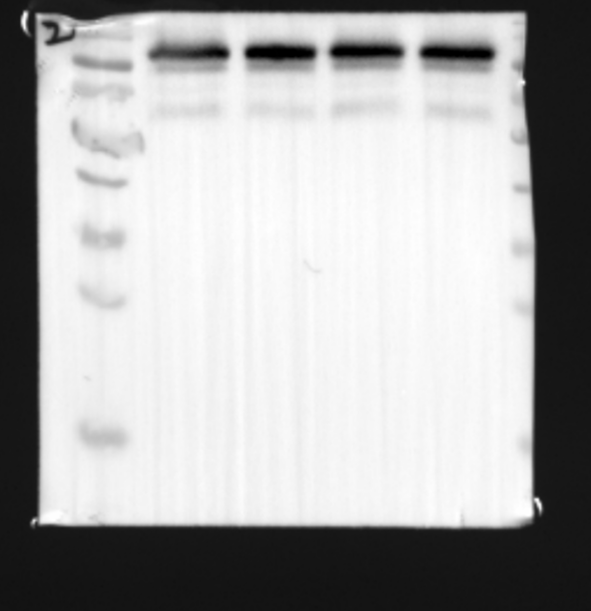

Supplement: Figure 5—source data 2. [file elife-108995-fig5-data2.zip › Figure 5, Source Data 2/PSD95 (K).tif]

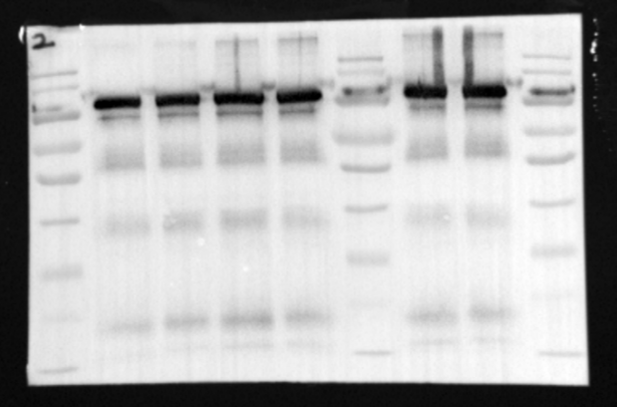

Supplement: Figure 5—source data 2. [file elife-108995-fig5-data2.zip › Figure 5, Source Data 2/PSD95 (N).tif]

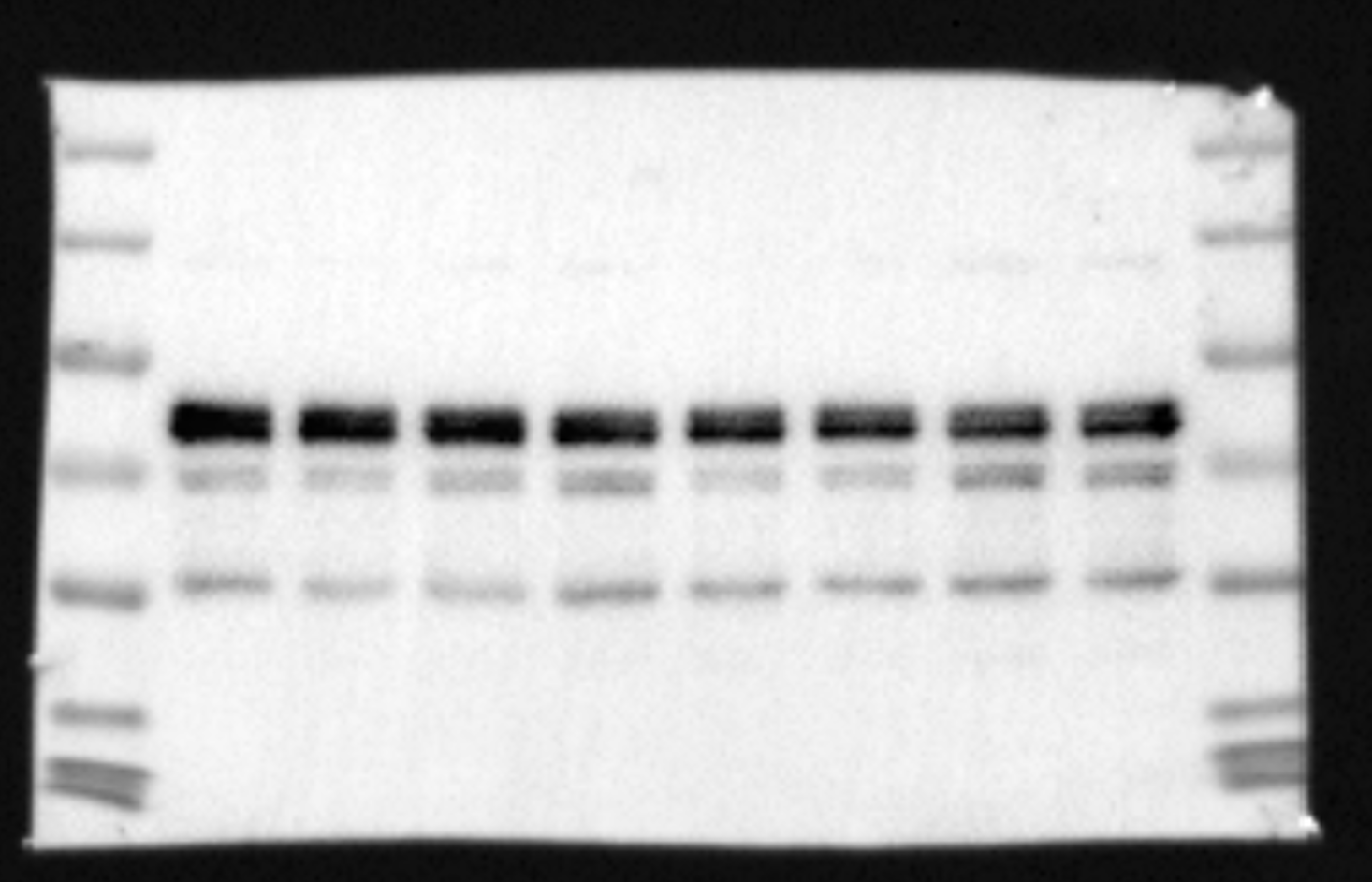

Supplement: Figure 5—source data 2. [file elife-108995-fig5-data2.zip › Figure 5, Source Data 2/SYN1 (B).tif]

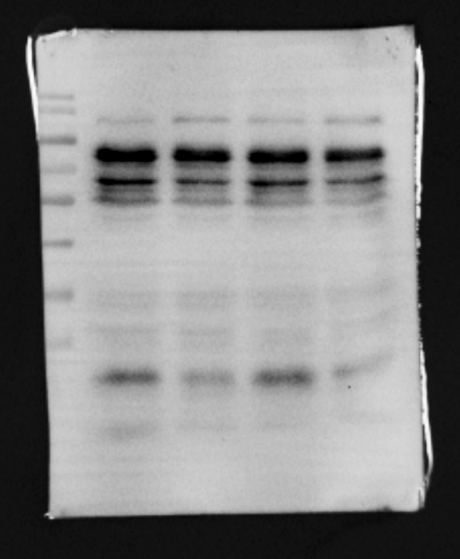

Supplement: Figure 5—source data 2. [file elife-108995-fig5-data2.zip › Figure 5, Source Data 2/SYN1 (C).tif]

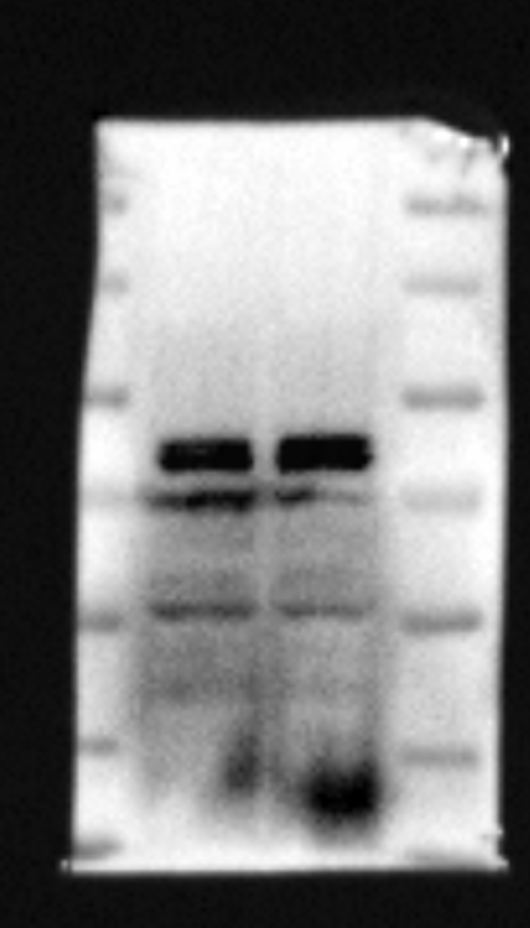

Supplement: Figure 5—source data 2. [file elife-108995-fig5-data2.zip › Figure 5, Source Data 2/SYN1 (D).tif]

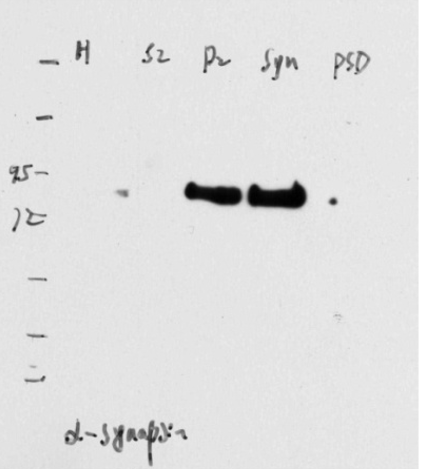

Supplement: Figure 5—source data 2. [file elife-108995-fig5-data2.zip › Figure 5, Source Data 2/SYN1 (H).tif]

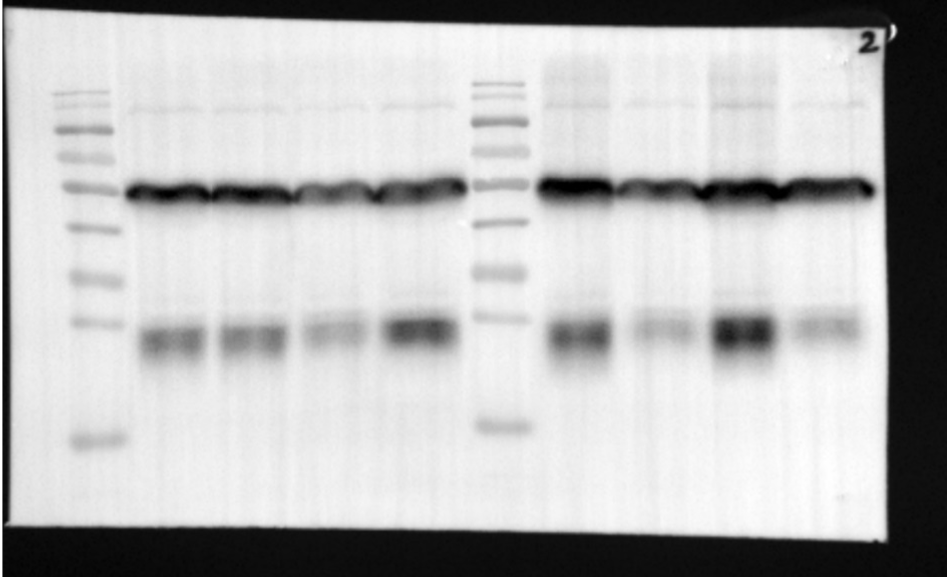

Supplement: Figure 5—source data 2. [file elife-108995-fig5-data2.zip › Figure 5, Source Data 2/α-tubulin (F).tif]

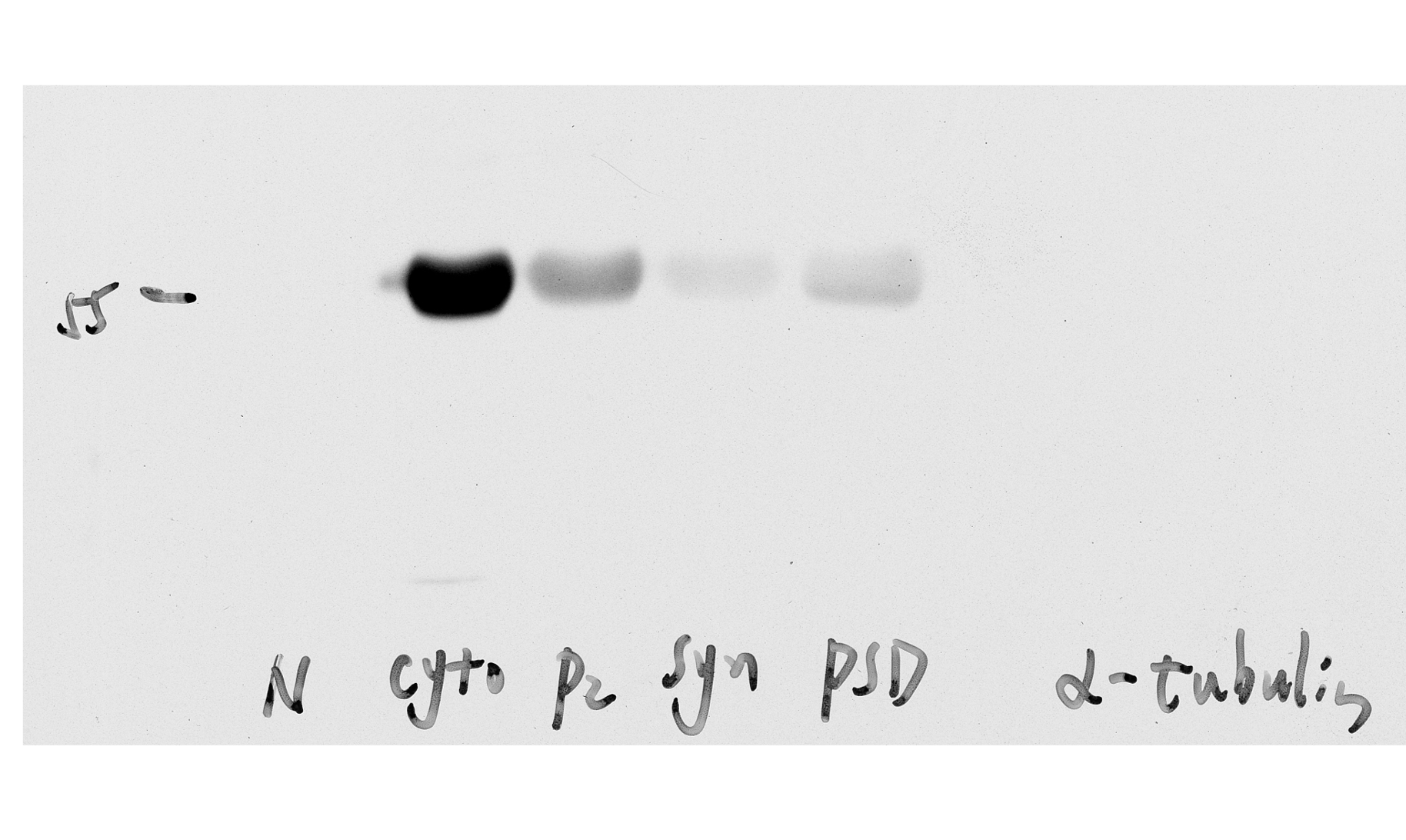

Supplement: Figure 5—source data 2. [file elife-108995-fig5-data2.zip › Figure 5, Source Data 2/α-tubulin (H).tif]

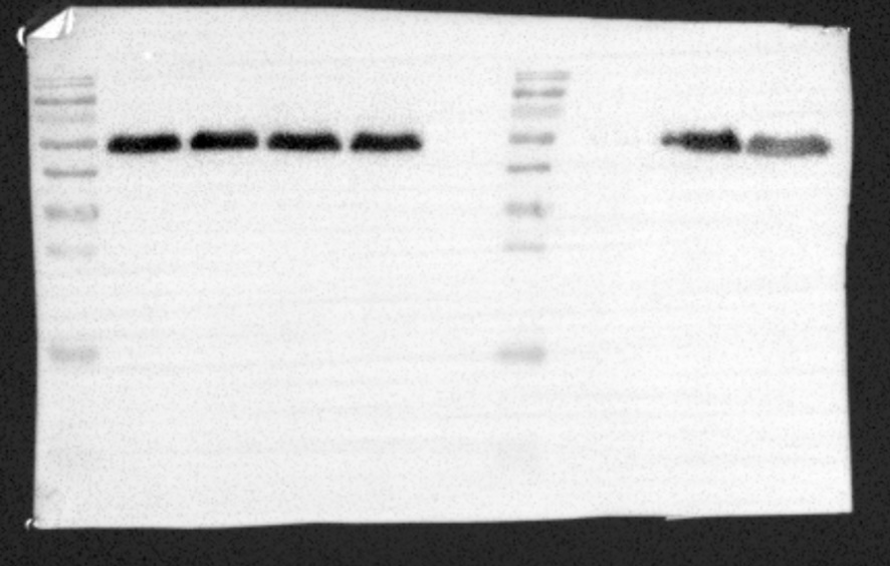

Supplement: Figure 5—source data 2. [file elife-108995-fig5-data2.zip › Figure 5, Source Data 2/α-tubulin (J).tif]

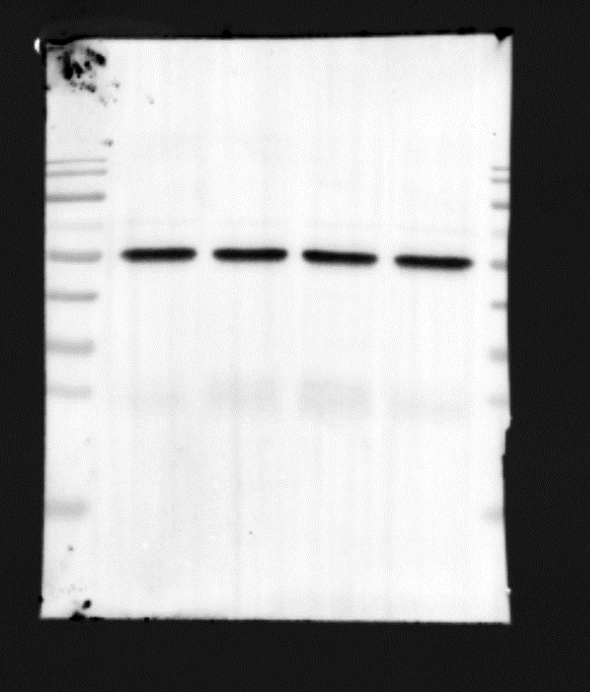

Supplement: Figure 5—source data 2. [file elife-108995-fig5-data2.zip › Figure 5, Source Data 2/α-tubulin (M).tif]

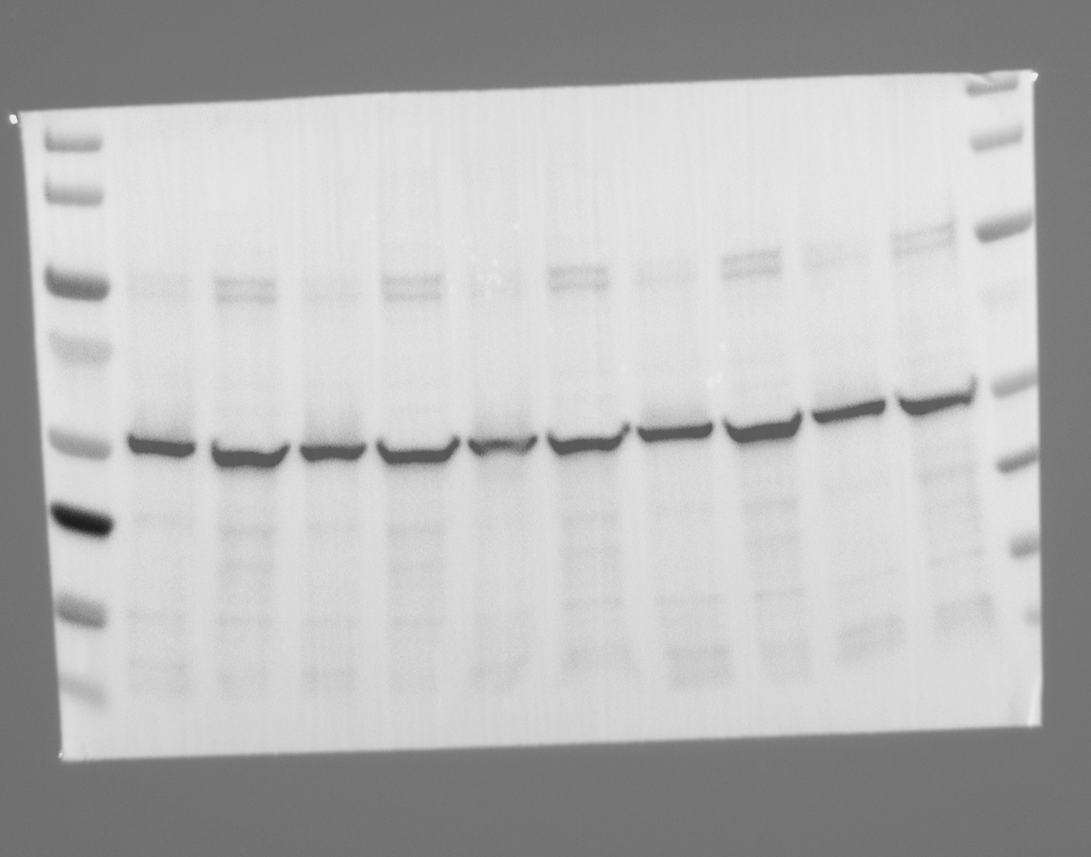

Supplement: Figure 5—figure supplement 1—source data 2. [file elife-108995-fig5-figsupp1-data2.zip › Figure 5, figure supplement 1, Source Data 1/arc.tif]

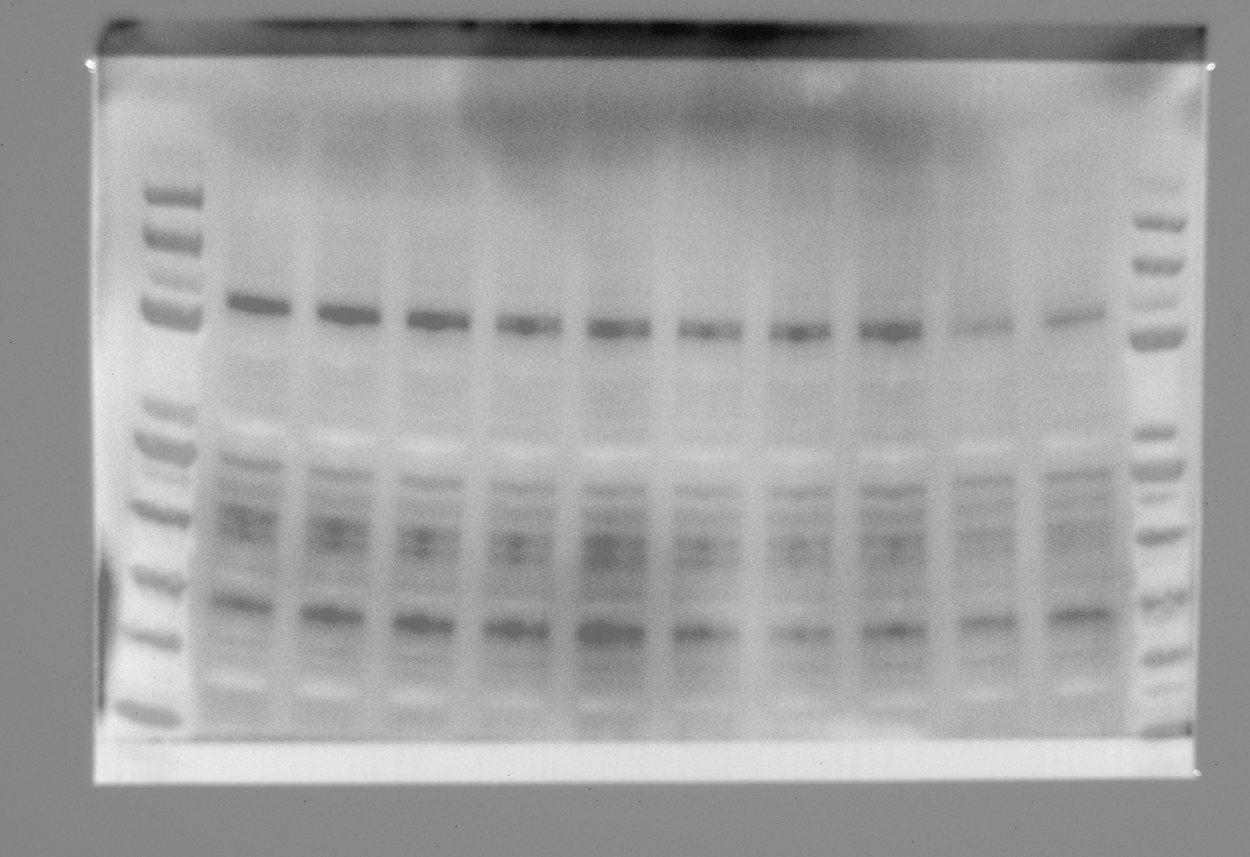

Supplement: Figure 5—figure supplement 1—source data 2. [file elife-108995-fig5-figsupp1-data2.zip › Figure 5, figure supplement 1, Source Data 1/SYN1.tif]

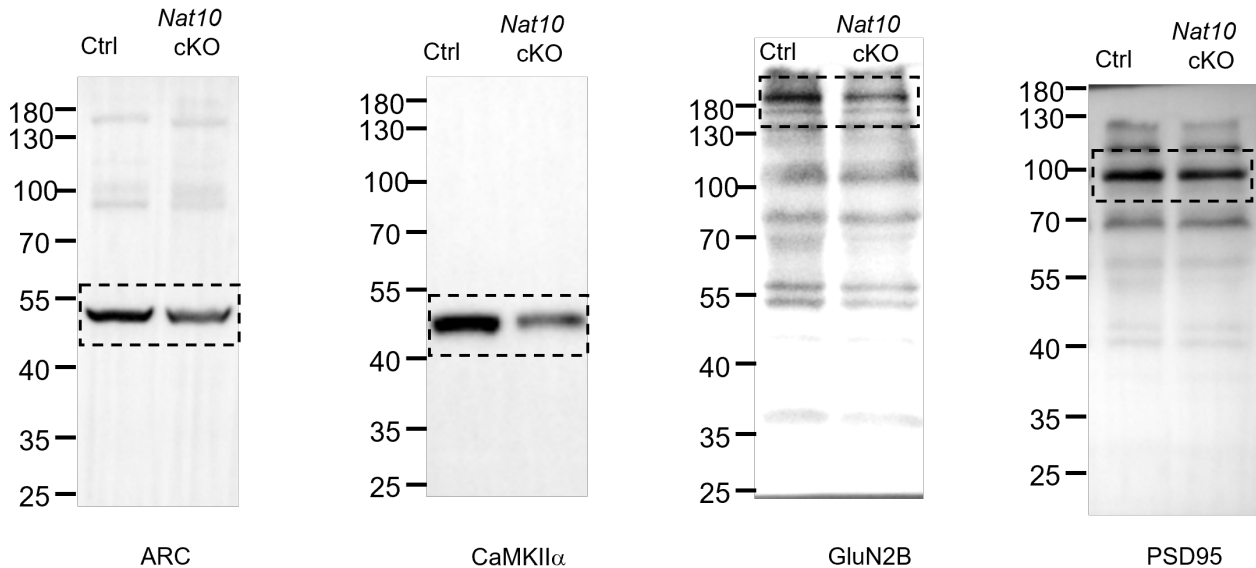

**Figure 6, Source Data 1.** Original membranes corresponding to Figure 6, panel J.

Supplement: Figure 6—source data 1. [file elife-108995-fig6-data1.zip › Figure 6, Source Data 1.pdf]

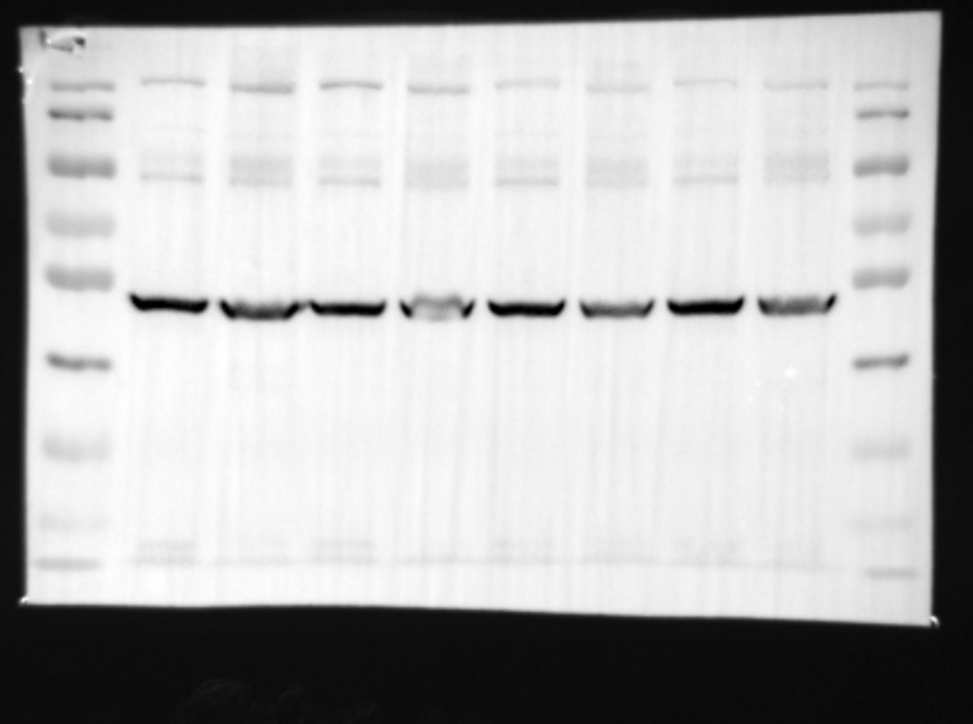

Supplement: Figure 6—source data 2. [file elife-108995-fig6-data2.zip › Figure 6, Source Data 2/ARC.tif]

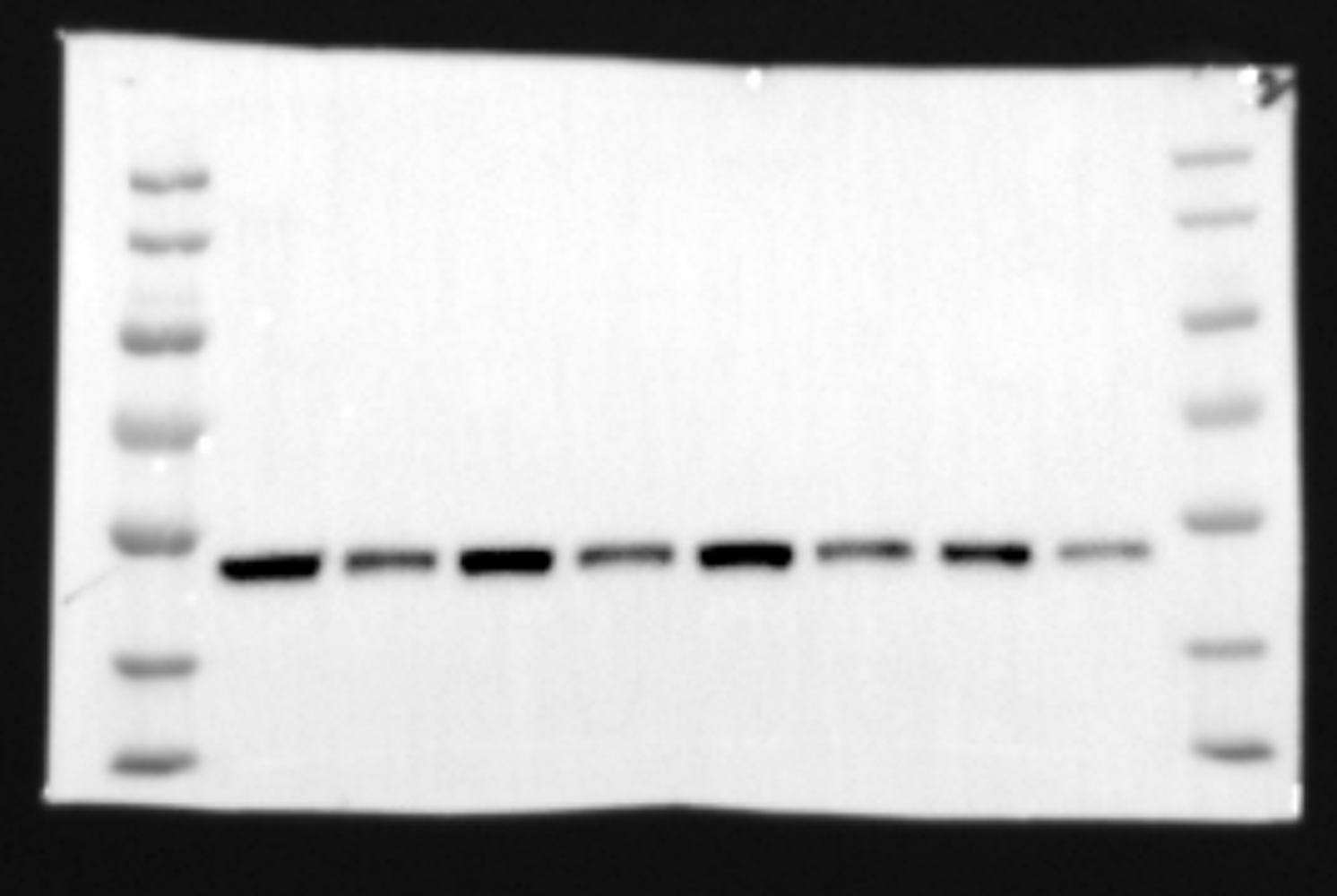

Supplement: Figure 6—source data 2. [file elife-108995-fig6-data2.zip › Figure 6, Source Data 2/CaMKIIa.tif]

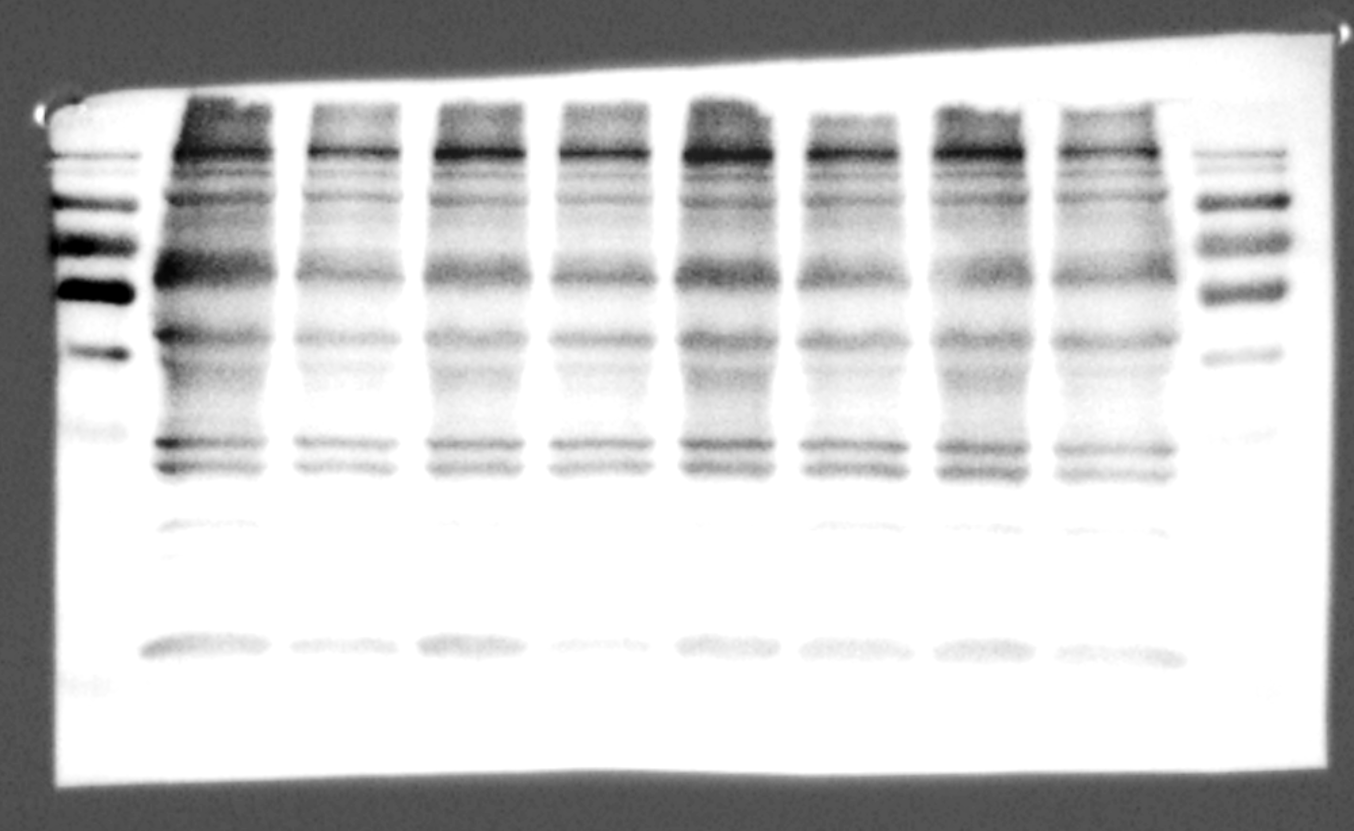

Supplement: Figure 6—source data 2. [file elife-108995-fig6-data2.zip › Figure 6, Source Data 2/GluN2B.tif]

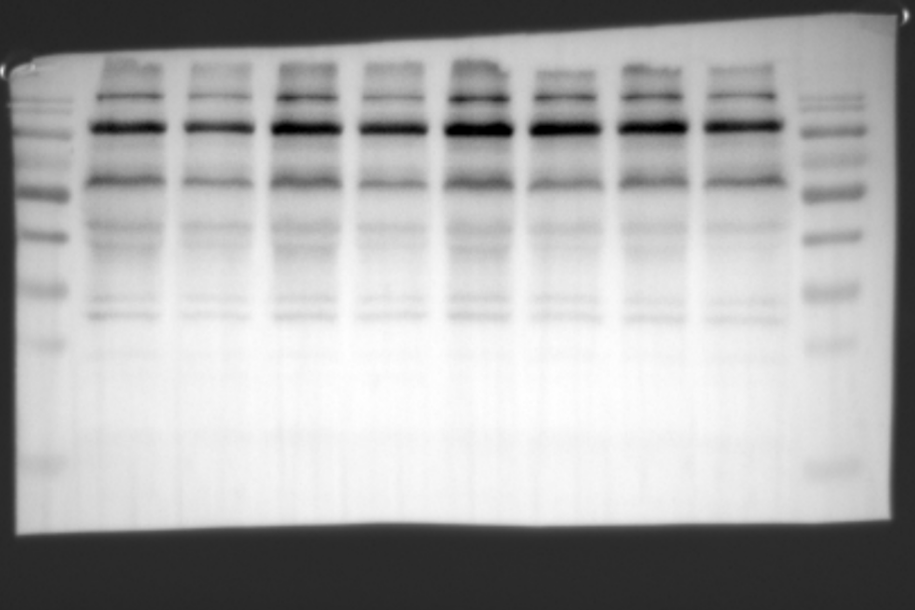

Supplement: Figure 6—source data 2. [file elife-108995-fig6-data2.zip › Figure 6, Source Data 2/PSD95.tif]

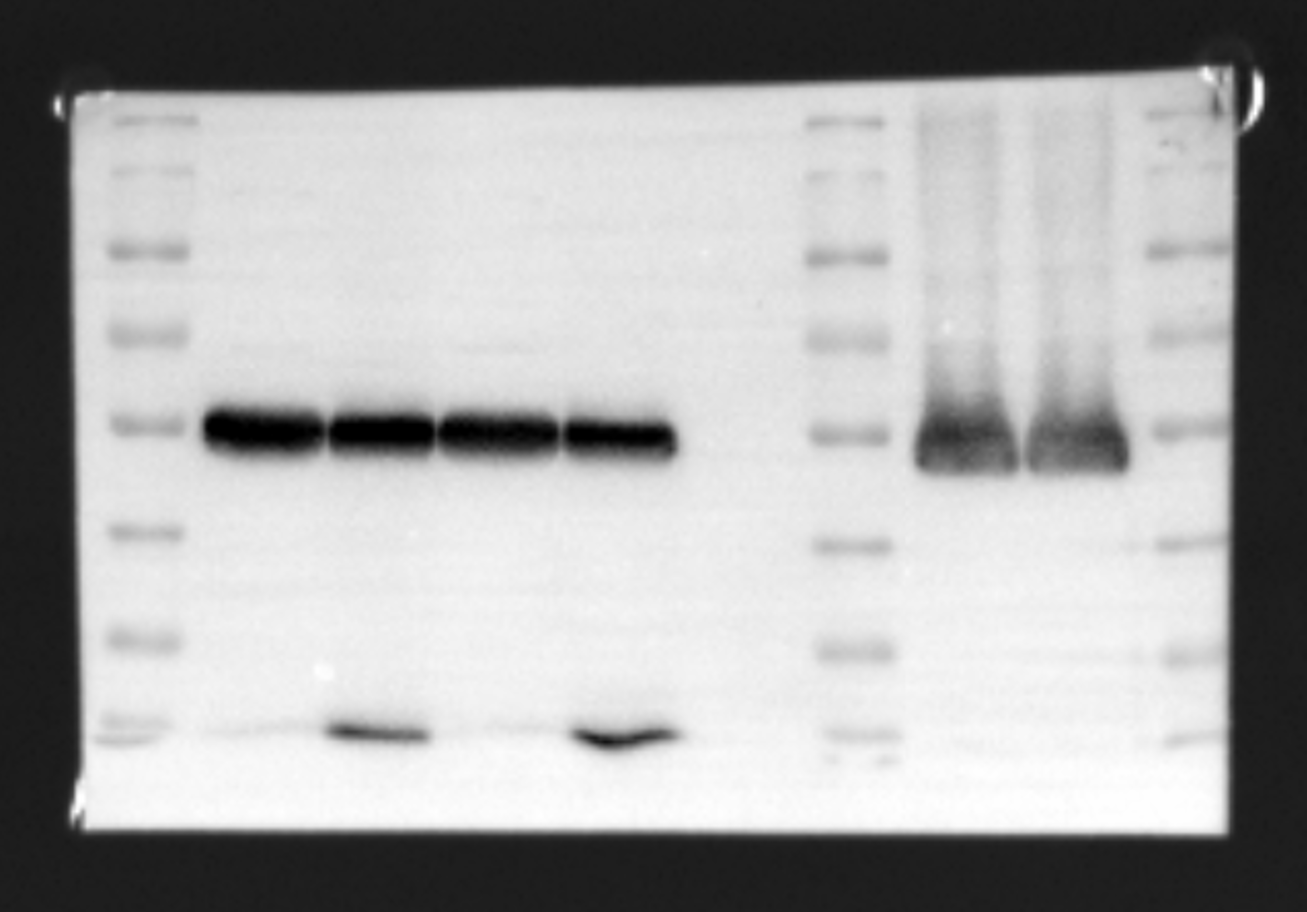

Supplement: Figure 6—figure supplement 1—source data 2. [file elife-108995-fig6-figsupp1-data2.zip › Figure 6-figure supplement 1, Source Data 2/a-tubulin.tif]

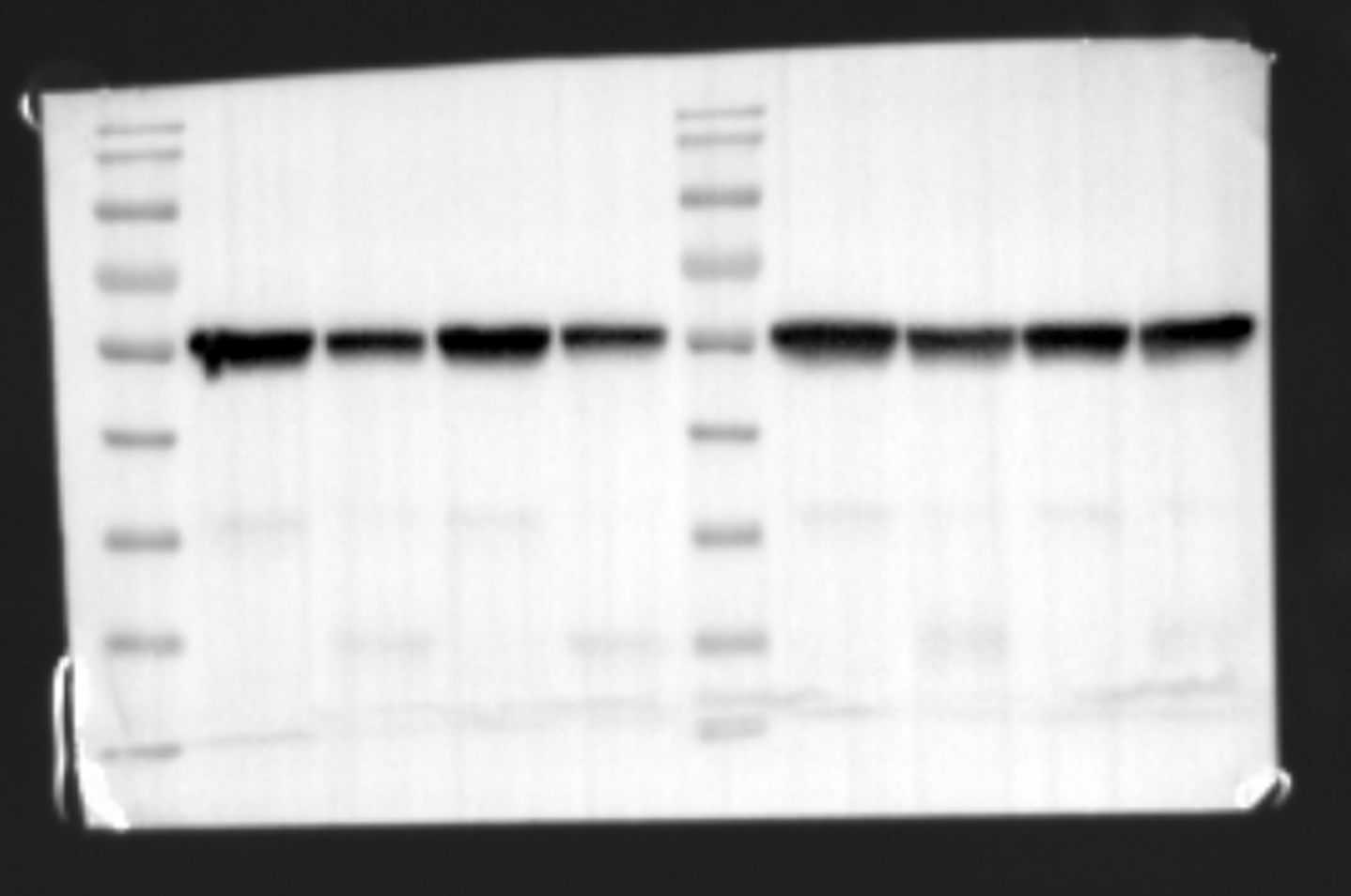

Supplement: Figure 6—figure supplement 1—source data 2. [file elife-108995-fig6-figsupp1-data2.zip › Figure 6-figure supplement 1, Source Data 2/Ac-a-tubulin.tif]

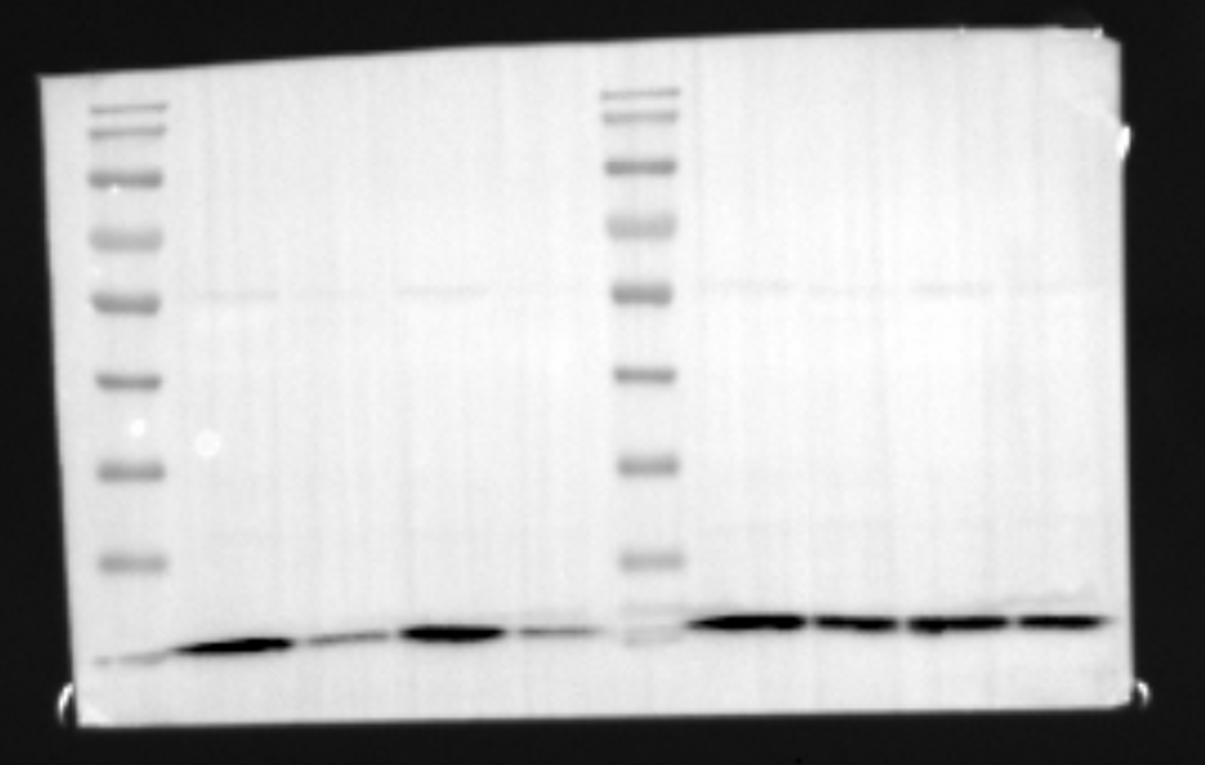

Supplement: Figure 6—figure supplement 1—source data 2. [file elife-108995-fig6-figsupp1-data2.zip › Figure 6-figure supplement 1, Source Data 2/Ac-H3.tif]

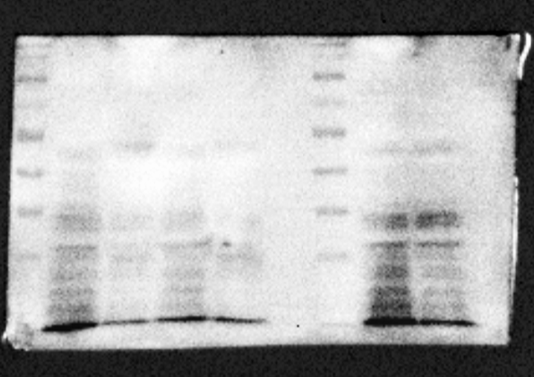

Supplement: Figure 6—figure supplement 1—source data 2. [file elife-108995-fig6-figsupp1-data2.zip › Figure 6-figure supplement 1, Source Data 2/Histone H3.tif]

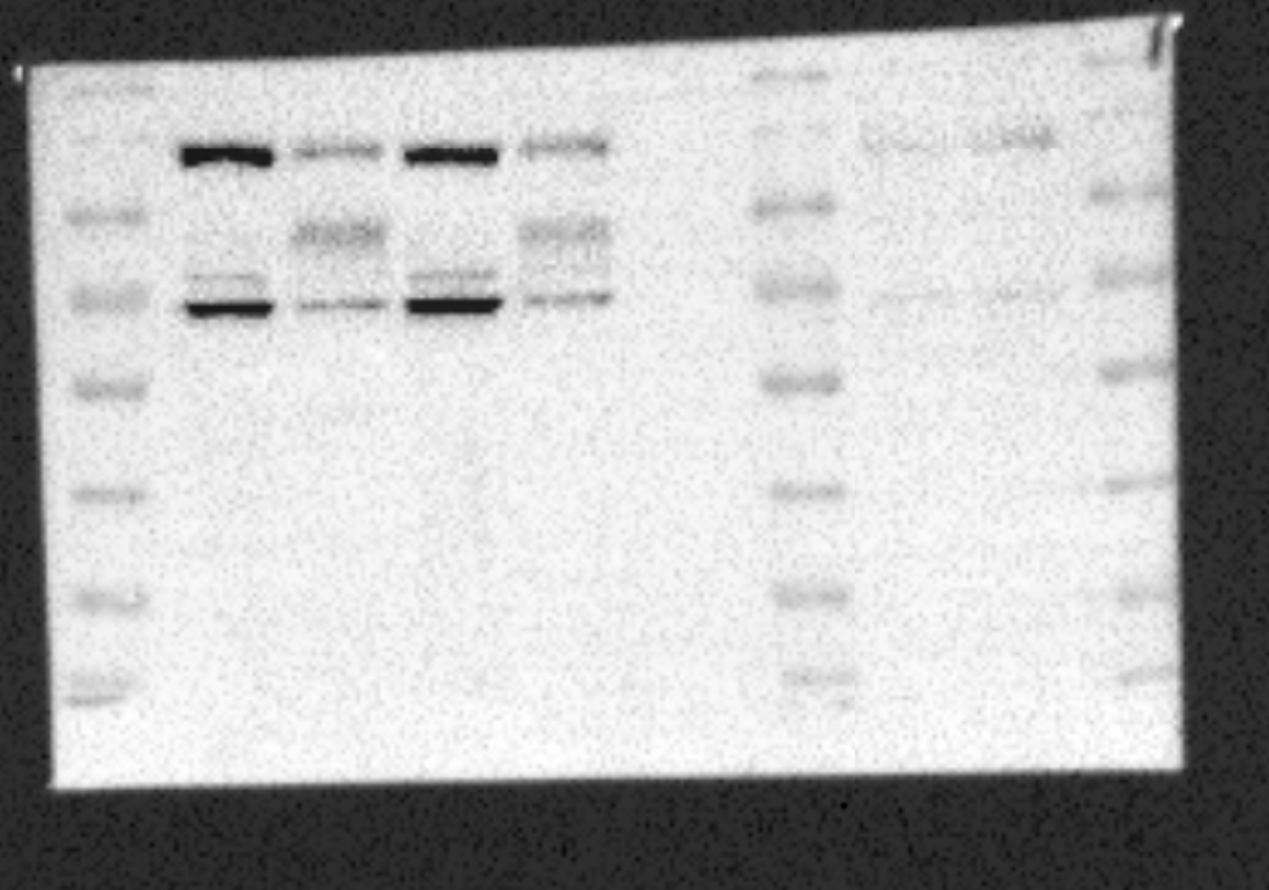

Supplement: Figure 6—figure supplement 1—source data 2. [file elife-108995-fig6-figsupp1-data2.zip › Figure 6-figure supplement 1, Source Data 2/NAT10.tif]
