# Supplementary material for: Dynamic regulation of mRNA acetylation at synapses by spatial memory in mouse hippocampus
Source: eLife. 2026 Mar 23;14:RP108995. doi: 10.7554/eLife.108995 (PMC13008358; doi:10.7554/eLife.108995)
Supplement: Figure 6—figure supplement 1—source data 1. [file elife-108995-fig6-figsupp1-data1.zip › Figure 6-figure supplement 1, Source Data 1.pdf]

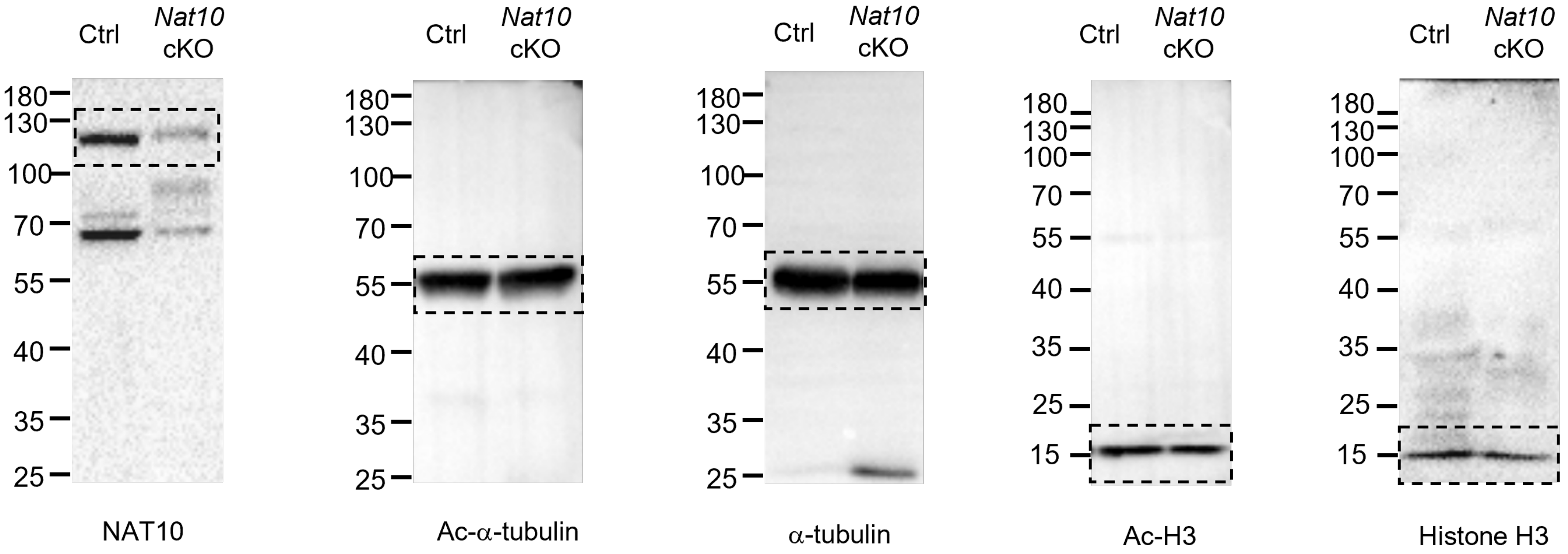

Figure 6-figure supplement 1, Source Data 1. Original membranes corresponding to Figure 6-figure supplement 1, panel C.
